# A computationally efficient quasi-harmonic study of ice polymorphs using the FFLUX force field

**DOI:** 10.1107/S2053273324010921

**Published:** 2025-01-01

**Authors:** Alexandra Pák, Matthew L. Brown, Paul L. A. Popelier

**Affiliations:** a Department of Chemistry University of Manchester Oxford Road Manchester M13 9PL United Kingdom; Universidad de Oviedo, Spain

**Keywords:** machine learning, quantum chemical topology, quasi-harmonic approximation, ice structures, polymorphism

## Abstract

FFLUX is a multipolar machine-learned force field that uses Gaussian process regression models trained on data from quantum chemical topology calculations. It offers an efficient way of predicting both lattice and free energies of polymorphs, allowing their stability to be assessed at finite temperatures. Here the I*h*, II and XV phases of ice are studied, building on previous work on formamide crystals and liquid water. A Gaussian process regression model of the water monomer was trained, achieving sub-kJ mol^−1^ accuracy. The model was then employed in simulations with a Lennard-Jones potential to represent intermolecular repulsion and dispersion. Lattice constants of the FFLUX-optimized crystal structures were comparable with those calculated by PBE+D3, with FFLUX calculations estimated to be 10^3^–10^5^ times faster. Lattice dynamics calculations were performed on each phase, with ices I*h* and XV found to be dynamically stable through phonon dispersion curves. However, ice II was incorrectly identified as unstable due to the non-bonded potential used, with a new phase (labelled here as II′ and to our knowledge not found experimentally) identified as more stable. This new phase was also found to be dynamically stable using density functional theory but, unlike in FFLUX calculations, II remained the more stable phase. Finally, Gibbs free energies were accessed through the quasi-harmonic approximation for the first time using FFLUX, allowing thermodynamic stability to be assessed at different temperatures and pressures through the construction of a phase diagram.

## Abstract 2

The next-generation machine-learning force field FFLUX is applied to ice polymorphs I*h*, II and XV. Under the quasi-harmonic approximation, Gibbs free energies are calculated using FFLUX at a significantly reduced computational cost compared with the commonly used density functional theory methods. However, the parametrized non-bonded potentials negatively affect the accuracy of the model, leading to large errors in the free energies calculated.

## 1. Introduction

Polymorphs are different crystalline structures of the same molecule, often varying in their physical properties and behaviour. The variation in properties means that in many industries (*e.g.* pharmaceuticals) it is crucial to avoid the formation of any unexpected polymorphs that could alter the properties of a product, such as an active pharmaceutical ingredient (Bauer *et al.*, 2001 ▸; Bučar *et al.*, 2015 ▸). Since experimental solid-form screening comes with a high cost, the use of computational crystal structure prediction (CSP) is becoming more prevalent. In these studies, between 10^5^ and 10^7^ potential structures are generated and optimized using cheap, low-accuracy methods. Around 10^2^–10^3^ of the lowest-energy structures are then taken forward to dispersion-corrected periodic density functional theory (DFT+D) calculations. DFT+D has been shown to significantly outperform traditional force fields (FFs) when it comes to finding experimentally observed structures in CSP studies (Reilly *et al.*, 2016 ▸; Day *et al.*, 2009 ▸; Hunnisett *et al.*, 2024 ▸), but it comes with a significant increase in computational cost, making state-of-the-art methods inefficient.

In CSP studies the relative stability of potential crystal structures is usually determined through lattice energies, with the most stable structure(s) assumed to be the one(s) with the lowest lattice energy. This approach neglects thermal contributions to the stability, which can be large enough to affect the energy ranking of phases as the relative energies of polymorphs can differ by as little as a few kJ mol^−1^ (Nyman & Day, 2015 ▸). Thermodynamic factors can be accounted for by calculating free energies using methods such as lattice dynamics (Pallikara & Skelton, 2021 ▸) or molecular dynamics (MD) (Hellman *et al.*, 2013 ▸), increasing computational costs even further.

To include free energies in CSP studies, we therefore require an efficient method that can reach quantum mechanical accuracy. One such method is the next-generation machine-learned force field FFLUX (Popelier, 2015 ▸; Symons *et al.*, 2021 ▸). FFLUX utilizes Gaussian process regression (GPR) models to predict the atomic energies and multipole moments of systems using data obtained from quantum chemical topology (QCT) calculations. Two QCT methods are important in the training of GPR models for FFLUX. The first method is the quantum theory of atoms in molecules (QTAIM) (Bader, 1985 ▸) which partitions the electron density into objects called topological atoms. The second is the interacting quantum atoms (IQA) energy decomposition scheme (Blanco *et al.*, 2005 ▸), which finds chemically relevant energy contributions by integrating the one- and two-particle density matrices over the volumes of topological atoms defined by QTAIM.

The GPR models in FFLUX are used to predict atomic energies and multipole moments up to the hexadecapole moment. Prediction of atomic energies allows for simulations with flexible molecules, free from the typical harmonic approximations used in traditional force fields; in turn this allows for a representation of the potential energy surface (PES) that lies closer to quantum mechanics. This is a benefit as other multipolar force fields used in CSP, such as DMACRYS (Price *et al.*, 2010 ▸), typically rely on rigid-body approximations. These models also allow for multipole moments that change with the geometry of the system on-the-fly in MD simulations, which is a feature unique to the FFLUX force field.

FFLUX has previously been applied successfully to liquid water (Symons & Popelier, 2022 ▸), formamide dimers (Brown *et al.*, 2023*b* ▸) and to formamide crystals (Brown *et al.*, 2023*a* ▸) with moderate success. In the study of formamide crystals, lattice dynamics calculations were performed with FFLUX for the first time, giving access to the Helmholtz free energies of the α and β polymorphs. These calculations were performed 10^5^ times faster than DFT+D, thereby significantly reducing the computational cost required for free energy calculations whilst maintaining a similar accuracy to DFT, but Lennard-Jones parameters proved insufficient for the β phase.

While liquid water has previously been studied with FFLUX, ice has not. Ice represents a significant challenge given its large number of polymorphs, with 19 known phases making up a complex phase diagram (Gasser *et al.*, 2021 ▸). Indeed, the well used water model TIP4P (Jorgensen *et al.*, 1983 ▸) had to be adapted such that it could more accurately model ice polymorphs, resulting in the TIP4P/Ice model (Abascal *et al.*, 2005 ▸). Most ice polymorphs form hydrogen order–disorder pairs where the hydrogens display a fractional occupancy of their crystallographic positions, while the oxygens exhibit full occupancy. This phenomenon occurs due to orientational disorder in water molecules when liquid water undergoes a transition from a disordered phase to the corresponding ordered phase. This transition can come about with cooling, sometimes requiring the aid of an acidic or a basic dopant where the introduction of defects leads to favourable rearrangement of the hydrogen-bond network (Salzmann *et al.*, 2006 ▸). In all cases, the ice structures satisfy the Bernal–Fowler ice rules, which state that the molecules are oriented such that there is only one hydrogen atom between two adjacent oxygens, and one oxygen is bonded to four hydrogens *via* two covalent bonds and two hydrogen bonds (Bernal & Fowler, 1933 ▸).

**Figure 1:**
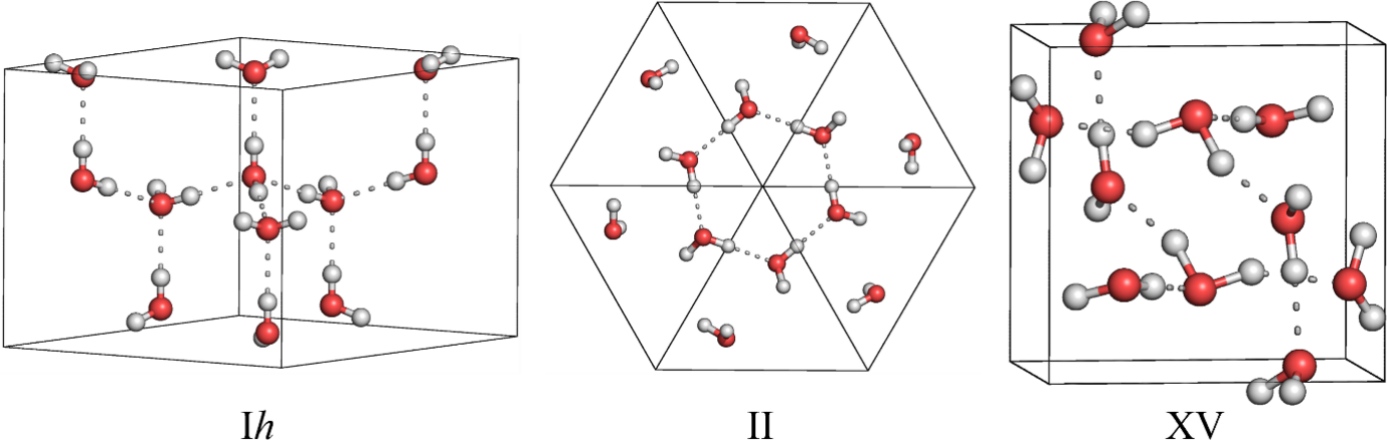
Experimental structures of ices I*h* (left), II (middle) and XV (right), studied in this work. Visualized using *PyMOL* (Schrodinger, 2015 ▸).

In this work, we apply FFLUX to ice I*h*, II and XV structures, which are shown in Fig. 1 ▸. With the exception of I*h*, these phases were chosen because they are ordered phases that are closely linked by pressure-based phase transitions. This work builds on the previous liquid water and formamide crystal studies, developing them further by now accessing the Gibbs free energies with FFLUX for the first time. These free energies are accessed through the quasi-harmonic approximation (QHA). The QHA has been used to study many systems, including determining phase diagrams for tin monochalcogenides from first principles (Pallikara & Skelton, 2021 ▸) as well as ices using the flexible q-TIP4P/F water model (Ramírez *et al.*, 2013 ▸) and MB-pol (Bore & Paesani, 2023 ▸). The validity of the QHA to study the free energies of ice polymorphs has previously been confirmed by comparing QHA calculations with thermodynamic integration (Vega *et al.*, 2008 ▸) and quantum path integral MD simulations (Ramírez *et al.*, 2012 ▸).

## 2. Methods

### 2.1. The FFLUX force field

The FFLUX force field is implemented in the *DL_FFLUX* code, which is built as a major add-on to *DL_POLY 4* (Todorov & Smith, 2018 ▸). Unlike the traditional bonded and electrostatic terms that classical FFs rely on, FFLUX utilizes GPR models to predict atomic energies and multipole moments, based on atoms defined *via* QCT. The theory behind FFLUX has been discussed extensively (Popelier, 2015 ▸; Symons *et al.*, 2021 ▸) but we briefly review it here.

#### 2.1.1. Quantum chemical topology

**Figure 2:**
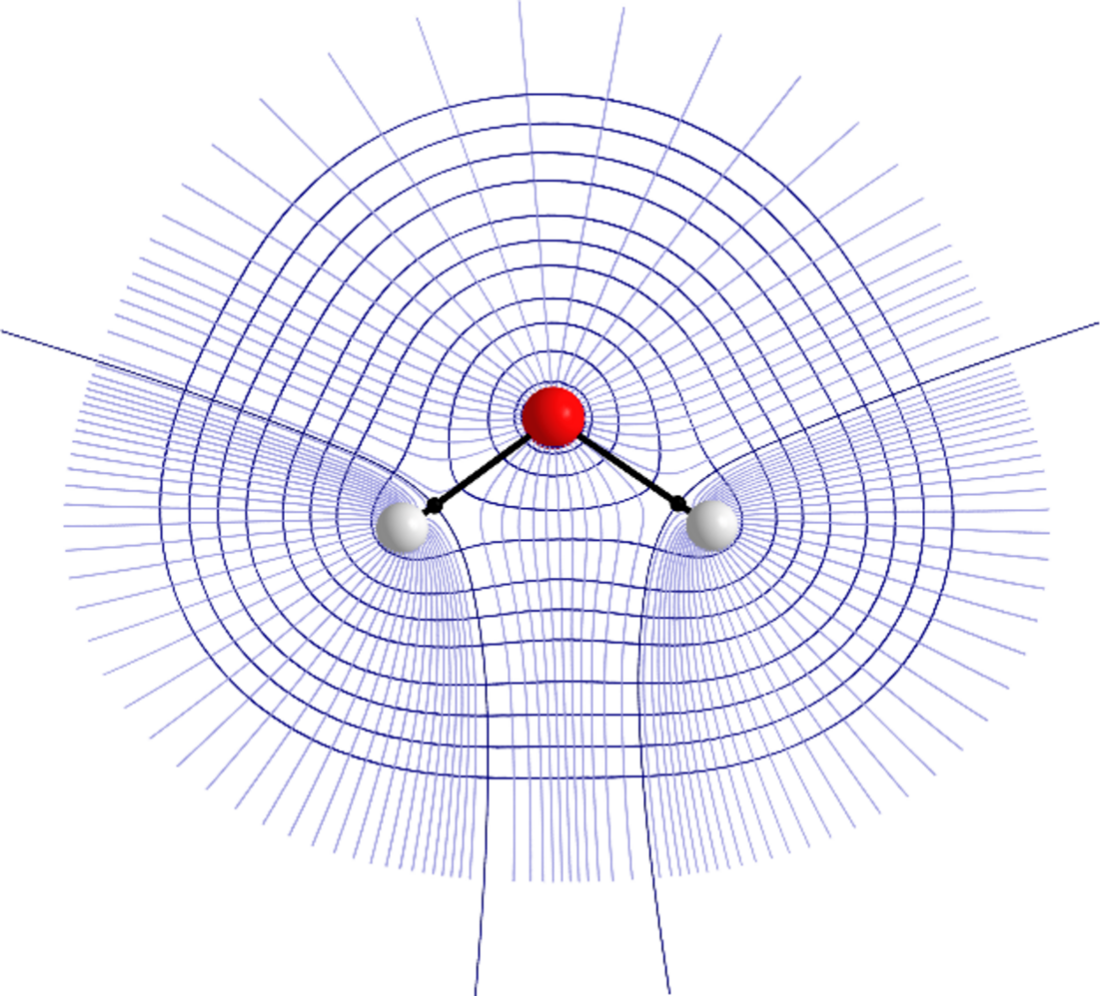
Contour plot of the electron density of the water monomer partitioned using QTAIM. Gradient paths are shown in blue, black paths represent the interatomic surfaces and black spheres represent bond critical points. This figure was made using *AIMStudio* (Keith, 2019 ▸).

QCT (Popelier, 2016 ▸) is a collection of methods that share the mathematical language of dynamical systems (*e.g.* attractor, basin, separatrix, critical point). QCT methods partition a quantum mechanical function using its gradient vector field. In the case of QTAIM, this function is the electron density. Applying the gradient vector field reveals so-called gradient paths running from infinity to critical points in the electron density. Maxima in the electron density are most often associated with nuclei and a collection of gradient paths terminating at these maxima form an object called a topological atom. A bond critical point (BCP) is a saddle point in the electron density, being a minimum in the direction of the bond path and a maximum in the two other directions. Gradient paths terminating at the BCP form a zero-flux or interatomic surface (IAS), with points on the surface obeying equation (1):
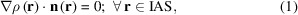
where 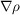 is the gradient of the electron density and 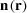 is a normal vector to a point 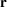 on the IAS. Topological atoms are non-overlapping and space-filling and hence have no gaps between them. Fig. 2 ▸ illustrates the partitioning of a water monomer.

While QTAIM is limited to stationary points on the PES due to the atomic virial theorem, the IQA partitioning scheme enables non-stationary geometries to be studied by calculating electron–electron potential energy terms independently from the kinetic energy term. The total energy of a system is then given by the sum of atomic energy contributions, 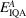. This energy can be partitioned further into intra- and interatomic energies, respectively, denoted 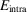 and 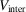:
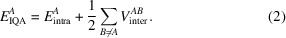


These intra- and interatomic energies associated with atoms *A* and *B* can be broken down further into chemically relevant contributions:
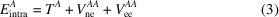

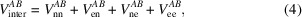
where n and e, respectively, indicate the nucleus or electrons belonging to atoms *A* and *B* depending on the matching subscript and superscript. The letter *V* refers to potential energy and *T* to kinetic energy (of electrons).


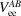
 can be further partitioned to give the Coulombic and exchange-correlation energies: 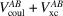. The ‘classical’, purely electrostatic, terms are then grouped together to yield 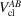, allowing us to express 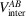 as
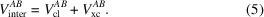


The Laplace expansion can be used to expand 1/*r*-type interactions. By expanding the 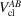 terms multipole moments can be generated, which express how the electron density is distributed. The atomic energies and multipole moments obtained from the IQA partitioning are used as the training data for the GPR models FFLUX uses in simulations.

#### 2.1.2. Gaussian process regression

GPR is a non-parametric Bayesian method (Rasmussen & Williams, 2006 ▸) used in FFLUX to predict atomic energies and multipole moments. The training data generated from IQA are mapped onto a set of geometries, allowing for flexible molecules in simulations as well as multipole moments up to the hexadecapole moment and atomic energies that change with the geometry, a feature unique to FFLUX.

The training set of the model is made up of input points, ***X***, containing *D* features in a set of *D*-dimensional input vectors. Each training point is associated with an output, *y*, collected in a vector, ***y***. Input features for the GPR models are described by the atomic local frame (ALF) (Konovalov *et al.*, 2021 ▸), a molecular representation that ensures the translational and rotational invariance of the models. Each atom, *A*, being trained for is the centre of its own local frame, with the highest-priority atom by Cahn–Ingold–Prelog rules, 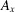, used to define the *x* axis and the second-highest-priority atom, 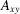, defining the *xy* plane. The *z* axis is constructed to form a right-handed axis system normal to this plane. The remaining atoms are described in spherical coordinates relative to the ALF. This means each model has 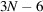 features, where the first two features are the 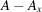 and 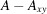 distances, and the third feature is the angle enclosed by the ALF atoms. In the case of terminal atoms, such as the hydrogen atoms in water, the 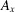 atom is determined as the only atom bonded to the atom of interest. The 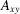 atom is then determined as the highest-priority atom by the Cahn–Ingold–Prelog rules bonded to atom 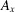 (Mills & Popelier, 2014 ▸).

In GPR the similarity between points is assessed using a covariance function. In the current work a radial basis function (RBF) kernel is used, modified to account for the periodic nature of every third feature, which has values ranging from −π to +π. This kernel is named the RBF-Cyclic kernel, and is shown in equation (6):
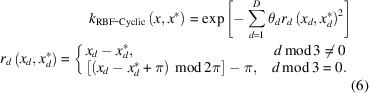
The distance between a feature, *d*, of training points **x** and 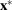 is scaled by a hyperparameter 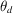, where *D* is the total number of features.

Model training involves finding the optimal set of these hyperparameters, which is done here using the iterative hold-out cross-validation (IHOCV) approach (Isamura & Popelier, 2023*b* ▸). This approach avoids drawbacks of the typically used type-II maximum likelihood approach, which is prone to propagating numerical errors, leading to inconsistent results (Isamura & Popelier, 2023*a* ▸). In the IHOCV protocol the θ values are obtained by minimizing the predictive root-mean-square error (RMSE) over an internal validation set. The RMSE cost function is given by equation (7):
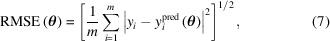
where *m* is the number of internal validation points, 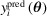 is the predicted value for the *i*th validation point (dependent on the model hyperparameters, 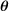) while 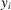 is the true value.

Following training, predictions are made by the GPR models according to equation (8):
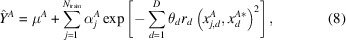
where 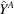 is the predicted multipole moment or energy of atom *A*, 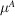 denotes the mean of the output over all training points, and 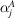 the ‘weight’ of training point *j*. The symbol 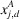 marks the *d*th feature of point *j* and 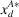 is the *d*th feature of the point to be predicted.

### 2.2. Lattice dynamics

While lattice energies are commonly used in the final ranking of potential polymorphs in CSP studies, more realistic rankings can be obtained through the calculation of free energies. One approach for calculation of free energies is through lattice dynamics calculations, which enable the study of the vibrations in solids, known as phonons. The computational cost associated with lattice dynamics calculations means that they are not routine in CSP studies despite the increased accuracy they offer (Reilly *et al.*, 2016 ▸).

The harmonic approximation (HA) is the fundamental level at which phonons can be modelled. Within the HA, the second-order interatomic force constant matrices 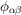 are given as
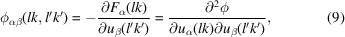
where 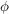 is the potential energy of the crystal, 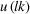 is the displacement of atom *k* in unit cell *l* and 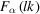 is the restoring force. The Cartesian directions *x*, *y* and *z* are indicated by subscripts α and β.

The dynamical matrix 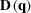 is then obtained by applying the Bloch theorem:
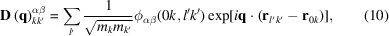
where 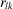 is the position of atom *k* in unit cell *l*, 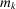 is the mass of atom *k* and **q** is a phonon wavevector in the Brillouin zone. For a crystal containing 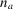 atoms in its unit cell, diagonalization of the dynamical matrix yields 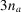 phonon frequencies, 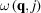, and corresponding displacement vectors, 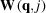, with band index *j*. Phonon frequencies can also be used to compute phonon dispersion curves, allowing the stability of a structure to be validated, and phonon density of states (DoS).

The thermodynamic partition function, *Q*, at a temperature, *T*, can be evaluated using the phonon frequencies according to equation (11):
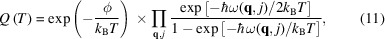
where 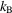 and 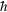 are the Boltzmann and reduced Planck constant, respectively. The Helmholtz free energy, *F*, is calculated from *Q* using the bridge relation:
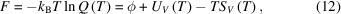
where 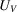 is the vibrational internal energy and 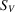 the vibrational entropy. Under the HA, interatomic forces are treated as purely harmonic and the volume dependence of phonons is not accounted for. However, this dependence can be studied using the QHA, which applies harmonic lattice dynamics calculations to a set of crystal structures with compressed and expanded volumes. The Gibbs free energy 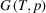 is then obtained as shown in equation (13):
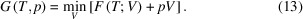


In practice, Gibbs free energies can be obtained by fitting the Helmholtz free energy as a function of volume, 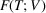, at each temperature *T* to an equation of state such as the Birch–Murnaghan equation of state (Birch, 1947 ▸),
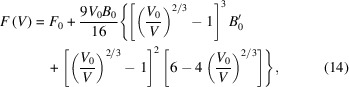
where 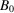 is the bulk modulus, 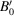 is the derivative of 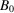 with respect to pressure, and 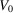 and 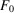 are the equilibrium volume and Helmholtz free energy, respectively.

## 3. Computational details

### 3.1. GPR models

For a FFLUX simulation, a GPR model is required for the energy of each atom in the molecule (or molecules) of interest as well as each component of its multipole moments (up to the hexadecapole moment). The dataset for the water monomer model was generated using our in-house Python pipeline *ICHOR* (Burn & Popelier, 2022 ▸; Manchev & Burn, 2024 ▸), and model training was performed with our in-house GPR engine *FEREBUS* (Di Pasquale *et al.*, 2016 ▸; Burn & Popelier, 2023 ▸; Isamura & Popelier, 2023*b* ▸).

Water geometries were generated in a 1 ns *AMBER* (Wang *et al.*, 2004 ▸) simulation with 1 fs timestep at 500 K. This temperature is higher than necessary for ices, but allows a larger domain space to be modelled which should improve the stability of the model. From the 1 million point trajectory, 10000 evenly spaced points were sampled and wavefunctions for each geometry were calculated at the B3LYP/aug-cc-pVTZ level of theory using *GAUSSIAN09* (Frisch *et al.*, 2010 ▸). The IQA partitioning was then carried out using *AIMAll* (Keith, 2019 ▸) to obtain atomic energies and multipole moments. The 10000 points were then filtered by recovery error, 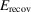, which represents the difference between the original wavefunction energy, 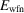, and the sum of the atomic energies from IQA,
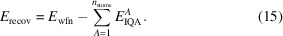
Geometries with an error greater than 1 kJ mol^−1^ were removed, reducing the dataset to 9998 points.

Stratified random sampling (SRS) (Isamura & Popelier, 2023*a* ▸) was used to sample the dataset and generate a training set (200 points), and internal and external validation sets (100 and 1000 points, respectively). The SRS method splits the data into subpopulations with an equal number of points, here with the use of the Freedman–Diaconis binning rule (Freedman & Diaconis, 1981 ▸), then points are selected randomly from the subpopulations. The internal validation set is used on-the-fly during model training for the optimization of the hyperparameters, while the external validation set is used to test the models. Hyperparameters were optimized using random update of the hierarchy ladder grey wolf optimizer (GWO-RUHL) (Isamura & Popelier, 2023*b* ▸) and the IHOCV protocol (Isamura & Popelier, 2023*a* ▸). Noise was added to the diagonal of the covariance matrix, with values optimized to range between 10^−6^ and 10^−14^.

### 3.2. Crystal structure optimizations

Due to the monomeric data on which they are trained, monomeric models cannot predict intermolecular dispersive and repulsive interactions, although this is possible within the FFLUX workflow (McDonagh *et al.*, 2018 ▸; Brown *et al.*, 2024 ▸), in principle and by proof-of-concept. In the current work, a straightforward 12–6 Lennard-Jones (LJ) potential was used to account for these interactions:
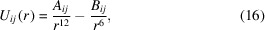
where 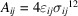 and 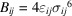, 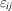 is the potential well depth and 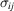 is the separation at which the potential energy between atoms *i* and *j* is zero.

As suggested by previous work (Brown *et al.*, 2023*a* ▸), in order to accurately model different polymorphs each phase would require a different set of non-bonded parameters. Given the large variance in the molecular environments of the different phases, it is expected that intermolecular interactions also significantly differ and therefore the same parameters will not be suitable for all phases. Details of how these parameters were obtained are given in Section S1 of the supporting information (SI), along with the parameters used in simulations.

Supercells of experimental structures were generated, with initial structures obtained from the Inorganic Crystal Structure Database (ICSD) (Hellenbrandt, 2004 ▸), as summarized in Table 1 ▸. Supercell expansions were selected such that electrostatic and van der Waals (vdW) cut-offs of 12 Å could be applied. The resulting supercell volumes were approximately 25^3^ Å^3^.

As *DL_FFLUX* is built as an add-on to *DL_POLY*, it can take advantage of several of the subroutines available in *DL_POLY*, such as the zero Kelvin (0 K) optimizer. In simulations using the 0 K optimizer, atoms are moved in the direction of their computed forces but not allowed to gain a velocity greater than they would at 10 K, effectively forcing the simulation into a local minimum. FFLUX optimizations of the crystal structures were performed using the 0 K optimizer in three stages. In the first stage an *NVT* ensemble was used, where only the atoms were allowed to move with the cell lengths and angles fixed. The final structure in this optimization was then used as a starting point for the second stage, where an *NPT* optimization was performed, allowing the cell lengths to change along with atomic positions. The final structure from this optimization was used as the initial structure in the third stage: an *N***σ***T* optimization, where the stress tensor and temperature were kept constant, also enabling cell angles to change.

Each stage had 7000 steps and a 1 fs timestep and was performed at 1 atm pressure. A Berendsen thermostat and barostat were used with a 0.2 ps and 0.5 ps relaxation time, respectively. For all phases, the optimizations were performed with an electrostatic rank of *L*′ = 2, where *L*′ denotes the highest multipolar rank present in the simulations. In the case of *L*′ = 2, electrostatic energies are calculated from the interactions between all multipole moments up to and including the quadrupole moment. Smooth particle mesh Ewald (SPME) (Essmann *et al.*, 1995 ▸) parameters were optimized with 10^−8^ precision.

Convergence of the optimizations was determined by looking at five gauges: (i) the maximum force component on the atoms, (ii) the root-mean-square (RMS) force, (iii) the maximum displacement component, (iv) the RMS displacement, and (v) the lattice energy. The optimized structure was determined as the timestep in the *N***σ***T* simulation, where the maximum and RMS forces acting on the atoms were below 2.2 kJ mol^−1^ Å^−1^ and 1.5 kJ mol^−1^ Å^−1^, respectively, and the maximum and RMS displacements were below 1.8 × 10^−3^ Å and 1.2 × 10^−3^ Å, respectively. Finally, the absolute difference in lattice energy between steps also had to be below 2 × 10^−5^ kJ mol^−1^.

### 3.3. DFT calculations

The unit cells obtained from the ICSD were also used as initial structures in optimizations using the *Vienna Ab initio Simulation Package* (*VASP*) (Kresse & Hafner, 1993 ▸). Electron exchange and correlation were modelled using the PBE (Perdew *et al.*, 1996 ▸) functional with the D3 (Grimme *et al.*, 2010 ▸) dispersion correction (PBE+D3). Core electrons were modelled using projector augmented-wave (PAW) pseudopotentials (Blöchl, 1994 ▸; Kresse & Joubert, 1999 ▸) with H 1*s* and O 2*s*/2*p* electrons in the valence shells. Structures were optimized to a tolerance of 10^−2^ eV Å^−1^ on the forces with a plane-wave cut-off energy of 850 eV and the Γ-centred **k**-point meshes given in Table 2 ▸. The plane-wave cut-off and **k**-point meshes were chosen such that the absolute total energies and pressures relative to the largest cut-off and mesh tested were converged to no more than 1 meV atom^−1^ and 1 kbar (0.1 GPa), respectively. The computational cost of optimizations using PBE+D3 and FFLUX is compared in Section S2.1 of the SI.

### 3.4. Lattice dynamics

Harmonic lattice dynamics calculations were performed on the FFLUX- and PBE+D3-optimized structures using the *Phonopy* package (Togo & Tanaka, 2015 ▸). A series of structures with single atoms displaced by 5 × 10^−3^ Å were generated from the optimized structures, and single-point force calculations were performed on each of them. In *VASP* calculations, the reduced **k**-point meshes and supercell expansions described in Table 2 ▸ were used in the single-point calculations, while FFLUX calculations used the supercell sizes described in Table 1 ▸. Unlike in previous work where the whole FFLUX-optimized supercell was used to generate displaced structures (Brown *et al.*, 2023*a* ▸), here a central unit cell was extracted, with the displaced supercells constructed using *Phonopy* for the force calculations. As up to 6*N* displacements may be required for *Phonopy* calculations, where *N* is the number of atoms in the cell provided to *Phonopy*, this procedure allows for a significant reduction in the number of required calculations proportional to the chosen supercell size. For example, in the case of ice I*h* the 4 × 4 × 4 supercell containing 2304 atoms required 13824 single-point calculations. However, using only the unit cell with 36 atoms meant just 216 calculations were required, a 64 times reduction (4 × 4 × 4 = 64) in the number of required calculations. The accuracy of extracting the unit cell compared with providing *Phonopy* with the FFLUX-optimized supercell is demonstrated in Section S2.2 of the SI. A comparison of the computational costs of FFLUX and PBE+D3 single-point force calculations is also provided in Section S2.3 of the SI.

Phonon dispersions were then generated to assess the dynamic stability of each phase, and the phonon DoS was evaluated by interpolating the phonon frequencies onto regular 16 × 16 × 16 **q**-point meshes. The relative stability of phases was then assessed by calculating the Helmholtz free energies.

The QHA extends on the HA, allowing a volume dependence of free energies to be calculated, therefore affording the Gibbs free energy. The volumes of optimized ice structures were compressed and expanded, then optimized with their volumes constrained to the scaled values. Harmonic phonon calculations were performed for each of the volume-scaled structures, the Gibbs free energy was then calculated over a 10–300 K temperature range as described in Section 2.2 using the *Phonopy* package.

## 4. Results and discussion

### 4.1. GPR model performance

Before applying the trained GPR models in a simulation, their predictive ability can be assessed. Initially, this can be done by plotting cumulative distributions of the absolute prediction error over the external validation set, called S-curves. The predicted properties of all points in the set are compared with the true values, organized in ascending order and then plotted against percentile. S-curves for the IQA energy and atomic charge are shown in Fig. 3 ▸, with S-curves for the remaining multipole moments provided in Section S3 of the SI (Figs. S2–S25).

The S-curves show that hydrogen energies are predicted with errors <0.1 kJ mol^−1^ across almost the entire test set, only showing greater errors above the 99% percentile. The oxygen atom has the largest errors, although these are also <0.1 kJ mol^−1^ for 97% of test points with the maximum error (at 100%) being just 1.18 kJ mol^−1^. This error is well below the commonly used 1 kcal mol^−1^ (4.18 kJ mol^−1^) threshold of chemical accuracy. Further assessment of the accuracy of the multipole moment models is provided in Section S3 of the SI (Fig. S26), where atom–atom intermolecular electrostatic interactions were predicted using the GPR model and compared with energies obtained from the IQA moments. The O–O intermolecular interaction is found to contribute most to the error, with an RMSE of only 0.016 kJ mol^−1^ across a validation set.

Geometry optimizations of the water monomer were performed using FFLUX to assess how well the minimum on the PES was reproduced. In the optimizations the 0 K optimizer was used in 5000-step simulations with a 1 fs timestep. The Berendsen thermostat with a 0.01 ps relaxation time was used. Fifteen distorted structures were generated by randomly varying the HOH angle and OH bond lengths of the B3LYP/aug-cc-pVTZ-optimized geometry. FFLUX consistently reproduced the optimized geometry of the training level of theory well, with an RMSE of 2.84 × 10^−4^ Å for all optimizations. Of note was that FFLUX was able to optimize an angle as large as 165° and bond lengths shorter or longer than equilibrium by 0.16 Å at maximum, with distorted structures covering an energy range of, at most, 148.7 kJ mol^−1^. These optimizations show that FFLUX is able to reproduce the minimum geometry with quantum mechanical accuracy. The energy of the optimized monomer was also captured with sub-kJ mol^−1^ accuracy, with FFLUX predicting an energy of −200761.94 kJ mol^−1^ compared with −200762.00 kJ mol^−1^ using the training level of theory, differing by only 0.06 kJ mol^−1^.

Additionally, the molecular dipole moment was calculated as 1.847 D with FFLUX, which is within less than 0.4% from the experimental value of 1.855 D (Clough *et al.*, 1973 ▸), and within 0.05% of the B3LYP-calculated value of 1.848 D. This demonstrates that the charge distribution in the molecule is reproduced with high accuracy. Further tests of the water GPR model are provided in Section S3 of the SI, showing its stability in MD simulations and its accuracy in vibrational frequency calculations. The model was found to be stable for at least 5 ns at multiple temperatures, and vibrational frequency errors corresponded to sub-0.1 kJ mol^−1^ energy differences from the training level of theory.

### 4.2. Optimized crystal structures

Following the process described in Section 3.2, the supercells of ices I*h*, II and XV were optimized with supercell expansions as shown in Table 1 ▸. Lattice constants of the FFLUX- and PBE+D3-optimized phases are shown along with the experimental values in Table 3 ▸.

FFLUX reproduces all experimental lattice parameters within 3%, without imposing any symmetry in the optimizations. These values are comparable with the PBE+D3 parameters. The similarity between the two methods is pleasing given the significantly shorter time required for the FFLUX optimizations (estimated to be on the order of 10^4^–10^5^ times faster than PBE+D3 in Section S2.1 of the SI). Moreover, the volume of I*h* is seen to decrease with respect to the experimental structure, consistent with thermal contraction. This effect can be seen in the optimizations as they are performed at 0 K and experimental structures are obtained at finite temperature. However, this is not the case for ices II and XV, which contract in the PBE+D3 but are seen to expand in the FFLUX optimizations. This expansion, while opposite to what is found with DFT, does have a physical basis given that II and XV are both high-pressure phases, and optimization under ambient pressure can lead to an expansion in cell volume.

Compared with PBE+D3, FFLUX does struggle to maintain the symmetry of the crystal. This difficulty can first be seen in the cell lengths, where the 1:1 ratio of *a*:*b* lattice parameters in I*h* and the 1:1:1 ratio of *a*:*b*:*c* in II are not maintained. Similar distortions are also seen in the cell angles. Determining the space group using *Phonopy* also indicates FFLUX’s struggle, with the experimental space groups of I*h*, II and XV recovered within 6 × 10^−2^, 3 × 10^−1^ and 3 × 10^−3^ Å in FFLUX-optimized structures. These values follow the trend of the space-group symmetry, with the highest-symmetry structure (ice II) having the largest error and the lowest-symmetry group (ice XV) having the smallest error. Errors in symmetry were not a problem with PBE+D3 calculations, with all experimental space groups recovered within 10^−5^ Å.

Optimization of potential structures is a key component of CSP studies, which then typically predict relative stabilities of structures using lattice energies. The lattice energies, 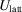, for the three optimized phases were calculated using
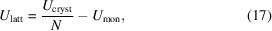
where 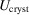 is the potential energy of the supercell, *N* is the number of molecules in the supercell and 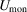 is the energy of an optimized monomer in the gas phase. The lattice energies are given in Table 4 ▸ as calculated by FFLUX and PBE+D3.

FFLUX predicts the lattice energy of phase I*h* to be remarkably close to experiment, differing by only 0.7 kJ mol^−1^, in contrast with PBE+D3 which predicts it to be more stable by 14.2 kJ mol^−1^. However, FFLUX calculates the energies of phases II and XV relative to I*h* to be much higher compared with PBE+D3 and experiment; instead of a sub-kJ mol^−1^ energy difference between I*h* and II, FFLUX predicts a difference of 18.6 kJ mol^−1^.

The monomeric model used in simulations was shown to be capable of sub-kJ mol^−1^ accuracy and FFLUX predicts the lattice energy of ice I*h* within 1 kJ mol^−1^ from experiment. Hence, discrepancies with the other two phases suggest that the LJ parameters were not suitable for ices II and XV. Secondly, because the model was trained on a monomer only, it does not capture intermolecular polarization. To account for both intermolecular polarization and van der Waals interactions within the FFLUX methodology, GPR models need to be trained on a cluster rather than on the monomer only, as done here. This work is already underway in our group (Brown *et al.*, 2024 ▸) but the full implementation is challenging and thus represents a longer-term goal.

### 4.3. Harmonic approximation

**Figure 4:**
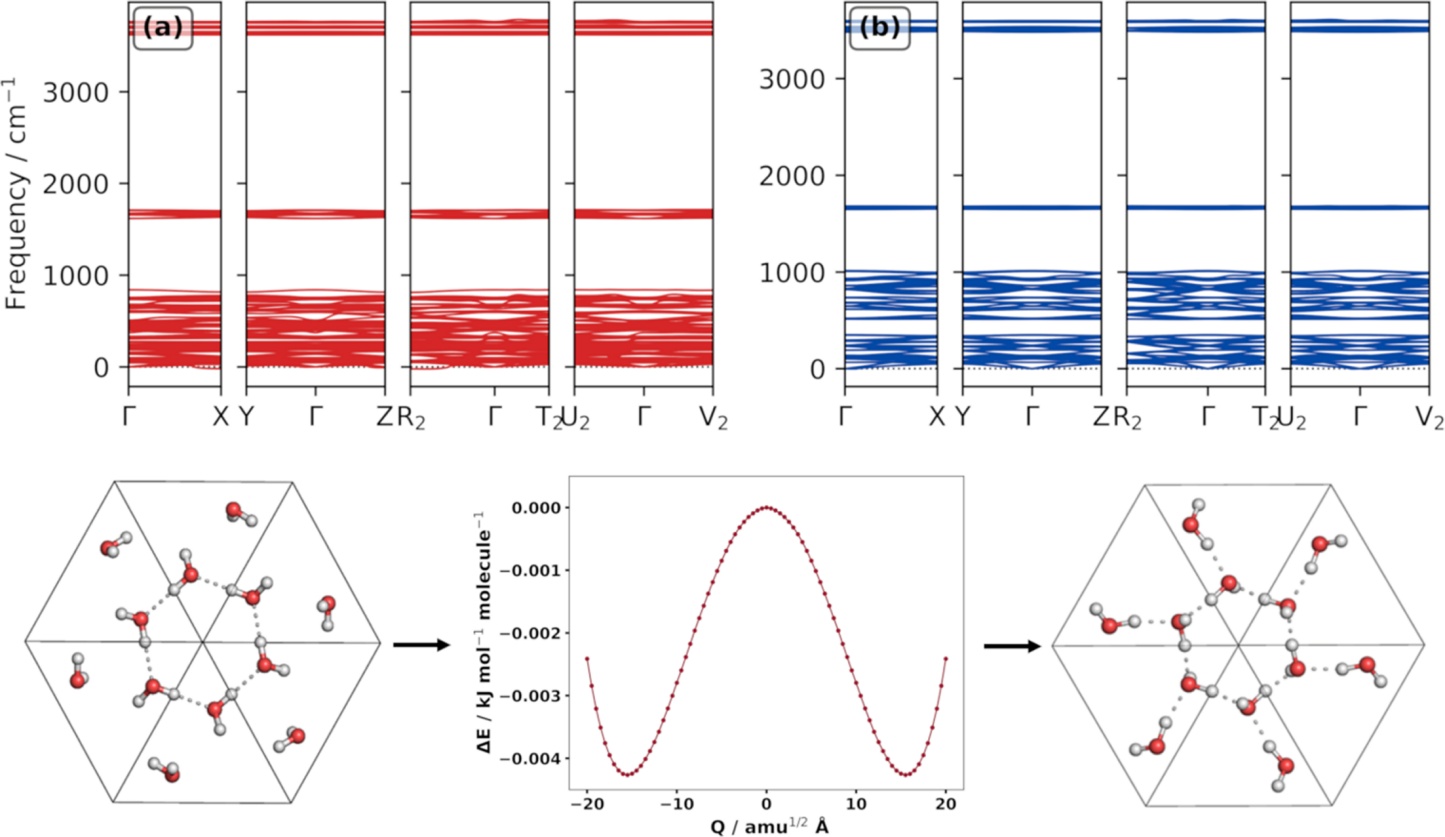
Phonon dispersion curve of (*a*) phase II, and of (*b*) phase II′. The unit-cell structure of II is shown at the bottom left, the mapped PES along wavevector 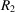 bottom middle and the structure of II′ at the bottom right.

Phonon frequencies, calculated from the process described in Section 2.2, were used to obtain phonon dispersion curves. The presence of imaginary phonon modes in the dispersion of a structure is indicative of dynamical instability (Pallikara *et al.*, 2022 ▸). Imaginary modes arise when the original structure is a maximum point on the PES and the displacement of atoms results in decreased energy. Phases I*h* and XV as optimized by FFLUX are considered dynamically stable, with their phonon dispersion curves given in Section S4.1 of the SI. However, ice II contains imaginary modes at high-symmetry points *X*, *Z* and 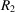, as shown in Fig. 4 ▸(*a*).

If imaginary phonon modes are present, the ‘mode-mapping’ technique can be used to investigate the instability (Skelton *et al.*, 2016 ▸). This process involves distorting the initial unstable structure along the imaginary mode eigenvectors with displacement amplitudes *Q*, generating a series of displaced structures. Calculating the energy of each distorted structure gives a double-well potential where the original geometry (at *Q* = 0) is a saddle point. The minimum-energy structure can then be optimized and tested for dynamical stability. The application of this approach has led to previously unknown polymorphs of bis­muth stannate (Rahim *et al.*, 2020 ▸).

The *ModeMap* code (Skelton *et al.*, 2016 ▸) was used to analyse the PES along wavevectors with imaginary phonon modes (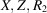) of ice II. Full details are given in Section S4.2 of the SI but the process is summarized in Fig. 4 ▸, where the 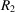 point was mapped to give a double-well potential. Optimization of the minimum of this potential resulted in a new structure that showed no imaginary modes [Fig. 4 ▸(*b*)], indicating dynamical stability. The arrangement of atoms and the lattice parameters of this new structure (here labelled II′) are different from those of the known ice II and, to our knowledge, from all other experimentally known ices. The unit cells of ice II and II′ are shown in Fig. 4 ▸ along with the mapped PES at point 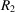. Further images are given in Section S4.2 of the SI.

To investigate whether ice II′ was an artefact of the non-bonded parameters used in the FFLUX calculations, DFT calculations were performed to optimize the structure and assess its stability in phonon calculations, as described in Sections 3.3 and 3.4 above. The phonon dispersion of II′ from PBE+D3 also shows no imaginary modes [Fig. S28(*d*)], confirming the dynamical stability of the structure outside of FFLUX parametrization. Lattice constants using both methods are similar, as shown in Table S5, but the II/II′ lattice energy ordering is different between methods, with PBE+D3 predicting II to be more stable by 1.9 kJ mol^−1^. Finally, PBE+D3 also finds ice II to be dynamically stable, meaning that no mode-mapping was required.

**Figure 5:**
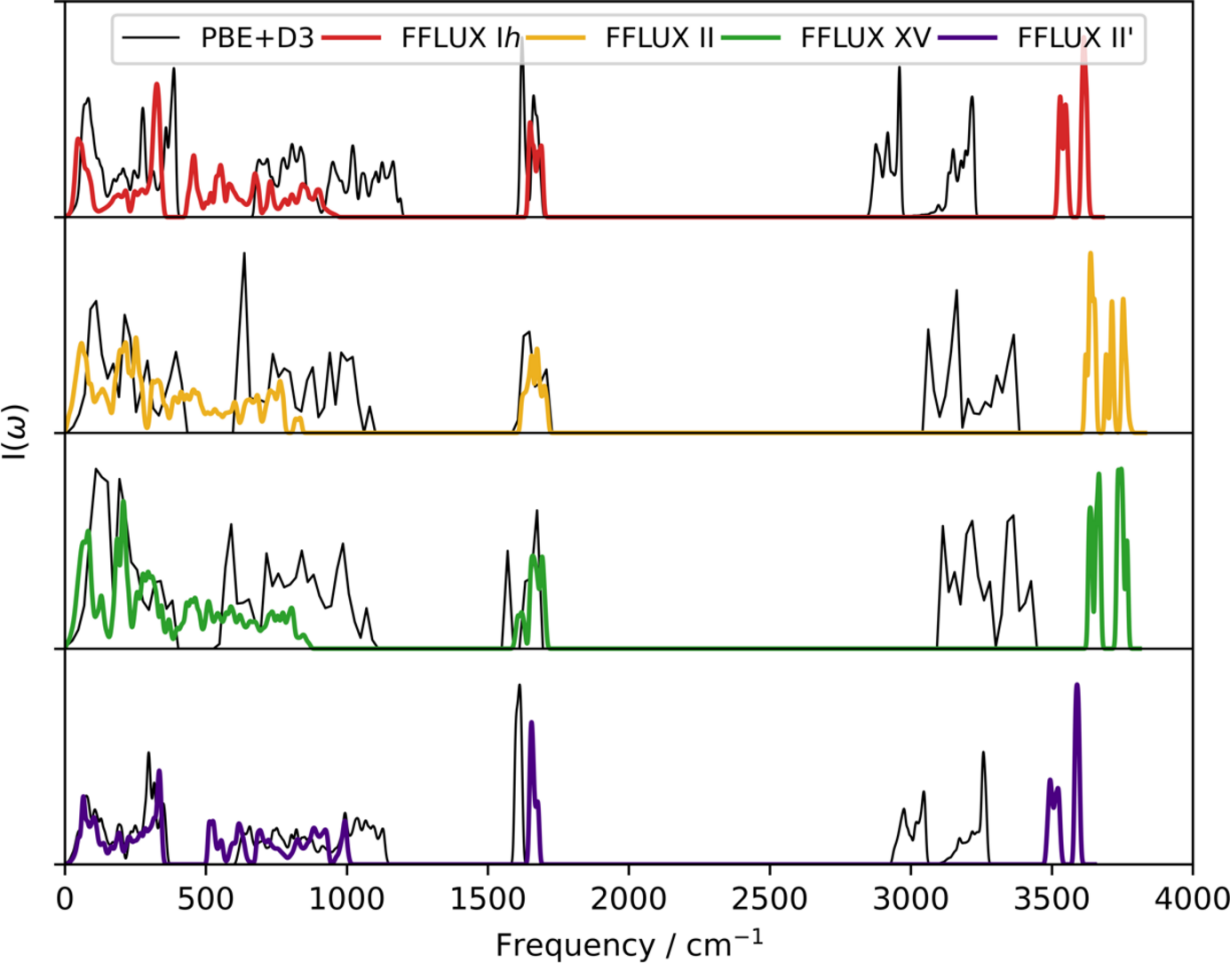
Phonon DoS of the studied phases calculated by PBE+D3 (black) and FFLUX (colour).

The previously calculated phonon frequency can also be used to plot the phonon DoS, shown in Fig. 5 ▸ for phases I*h*, II, XV and II′, calculated using PBE+D3 and FFLUX.

The high-frequency peaks corresponding to OH stretching modes observed in neutron scattering experiments at approximately 3100 and 3200 cm^−1^ (Prask *et al.*, 1972 ▸) in ice I*h* are significantly blue-shifted by FFLUX, while PBE+D3 predicts them to be more in-line with experimental data. These errors are counter to the highly accurate monomer vibrational frequencies presented in Table S4 of Section S3 of the SI. Differences between the two methods should be expected, with the GPR model used in FFLUX simulations being based on B3LYP/aug-cc-pVTZ calculations and the DFT calculations being PBE+D3/plane-wave. B3LYP is known to typically overestimate vibrational frequencies, requiring scaling factors to better represent experimental data (Sinha *et al.*, 2004 ▸). Furthermore, the monomeric nature of the model also contributes to the observed differences as intermolecular interactions that the GPR model currently cannot capture are known to red-shift (Dykstra, 1988 ▸) or blue-shift (Fornaro *et al.*, 2015 ▸) vibrational frequencies. The use of oligomeric models has been shown to improve the calculation of vibrational frequencies (Brown *et al.*, 2024 ▸) as they account for intermolecular interactions. The peak representing bending modes is observed at 1600 cm^−1^ experimentally in ice I*h.* This peak is captured well using FFLUX compared with PBE+D3 in the case of all four phases, near 1600 cm^−1^ by both methods.

**Figure 6:**
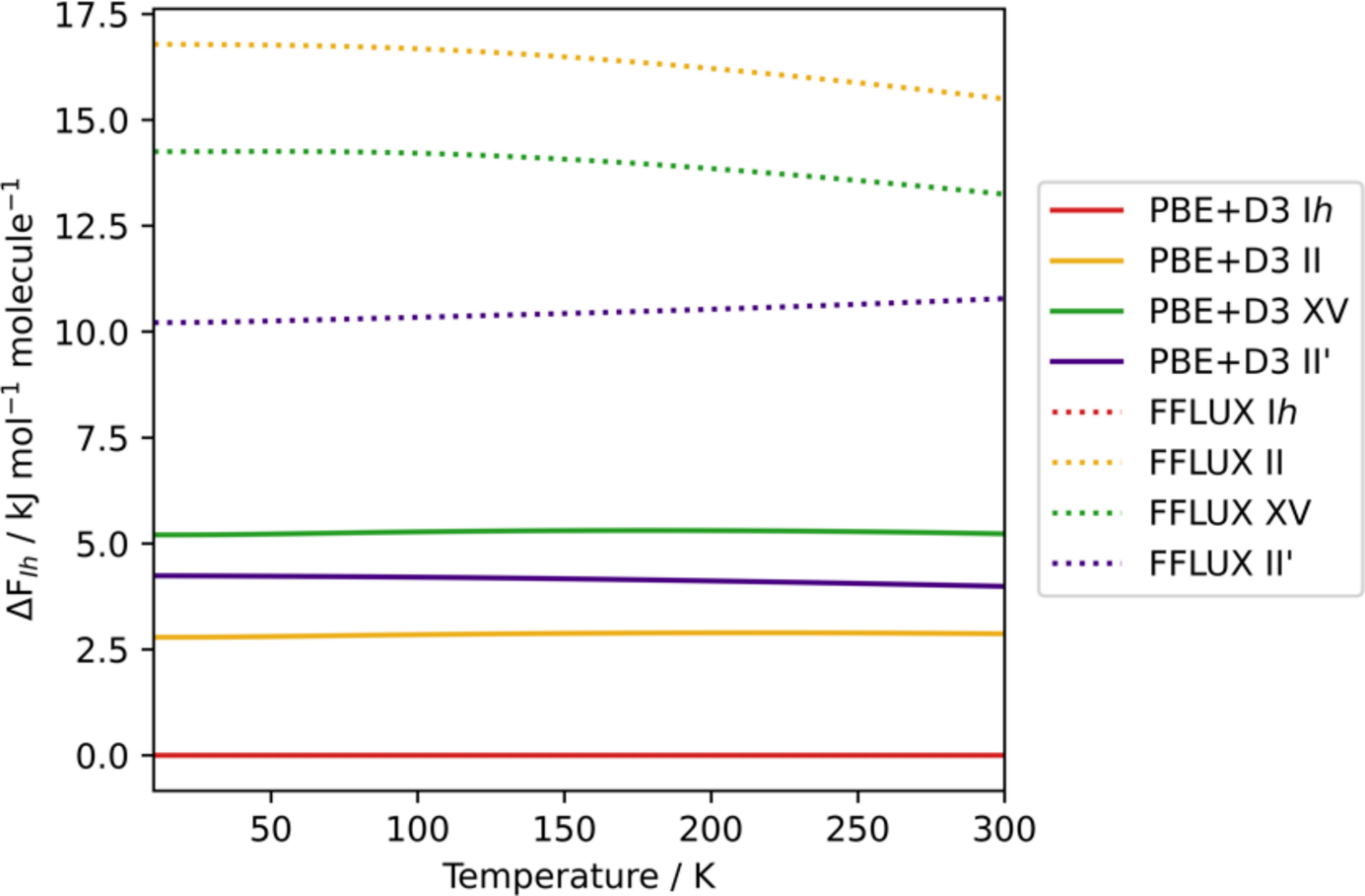
Helmholtz free energies of II, II′ and XV are shown relative to I*h*. Solid lines indicate PBE+D3-calculated energies, while dotted lines indicate calculations performed using FFLUX.

The lower-frequency regions contribute more to the free energy. FFLUX predicts these regions closest to PBE+D3 in the case of phase II′, reproducing the peaks below 500 cm^−1^ particularly well. The lowest region for I*h* is also predicted relatively well compared with PBE+D3 but the gap between the two peaks is significantly smaller. For phases II and XV, no gap is observed, as opposed to PBE+D3, and the peak is red-shifted. The errors in these frequencies possibly contribute to inaccuracies in the calculated Helmholtz free energies of the phases, shown relative to ice I*h* in Fig. 6 ▸.

The qualitative ordering of polymorphs with FFLUX is found to be mostly the same as that of PBE+D3, with ice II being the main discrepancy. The dynamic instability of II predicted by FFLUX is likely to be contributing to this mismatched result. Across the temperature range studied, both FFLUX and PBE+D3 predict phase I*h* to be most stable, with II′ being predicted as more stable than XV. Given that the polymorphs are related by pressure transitions, it is also pleasing to see the Helmholtz energies recover the expected monotropic behaviour of the polymorphs, meaning that no transitions are observed between phases across the temperature range.

Quantitatively, FFLUX predicts the relative energies to be much larger than those of PBE+D3. Given the accuracy of the monomeric model, this fact is likely due to the non-bonded parameters that were used. The transferability of the monomeric moments to a molecule in the crystal could also be contributing to the errors seen, as intermolecular polarization is not accounted for in the monomeric model.

### 4.4. Quasi-harmonic approximation

**Figure 7:**
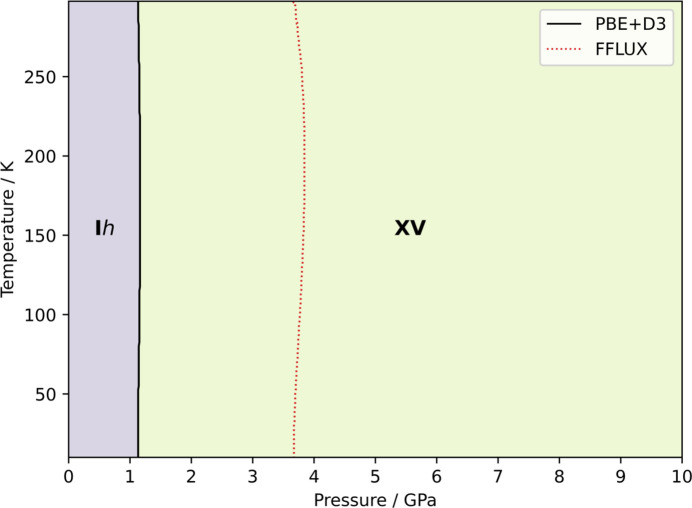
Phase diagram of phases I*h* and XV calculated by FFLUX and PBE+D3; the red dotted line indicates the pressure transition by FFLUX while the black solid line indicates that by PBE+D3.

To account for the volume dependence of phonon frequencies under the QHA, a series of volume compressions and expansions were introduced to the optimized structures followed by the application of the HA at each volume. Using PBE+D3 all structures were compressed and expanded by 10%. Using FFLUX, volume changes for ice I*h* were ±5%, and between −3 and +10% for ice XV. These volumes allowed for free-energy–volume curves that captured a minimum-energy structure enabling the QHA to be performed [see equation (13)]. We obtain Gibbs free energies at constant pressure, which are a more experimentally relevant way of comparing the stabilities. The Gibbs free energies calculated under the QHA can be used to construct a temperature–pressure phase diagram by adding a *pV* term to the free energies. Fig. 7 ▸ shows the I*h*/XV phase diagram, displaying the predicted behaviour over a 10–300 K temperature and 0–10 GPa pressure range using FFLUX and PBE+D3. Phase II has been omitted as FFLUX finds it to be metastable, as shown by the Gibbs-pressure plot in Fig. S34 in Section S4.2 of the SI.

Calculation of phase diagrams is a challenging test for any method (Ramírez *et al.*, 2013 ▸; Abascal *et al.*, 2005 ▸; Bore & Paesani, 2023 ▸), and it has been seen that errors in free energies as small as 1 kJ mol^−1^ can lead to transition temperature errors of over 200 K (Červinka & Beran, 2018 ▸), highlighting the high accuracy required for such calculations. Here, FFLUX predicts a pressure transition that is approximately 3.5 times that of PBE+D3, with the DFT calculations better representing the experimental transition where ice XV is seen to become stable at ∼0.8 GPa. Compared with other water models, FFLUX falls short here, with TIP4P/Ice showing errors that are of the order of 0.1 GPa (Abascal *et al.*, 2005 ▸). The MB-pol potential also reproduces the phase diagram closely (Bore & Paesani, 2023 ▸). However, it is quite common for phase diagrams to have large errors, with prediction being made easier for systems at high pressure, where thermal expansion effects become less significant (Beran, 2023 ▸).

Errors in the free energies calculated by FFLUX are likely to be due to the representation of dispersion and repulsion by a LJ potential. The way to circumvent the implementation of non-bonded parameters is by training on an oligomeric model, although incorporation into systems larger than the one we trained on is still under development.

## 5. Related literature

The following references are cited in the supporting information: Frisch *et al.* (2016 ▸), Fu *et al.* (2023 ▸), Hédin *et al.* (2016 ▸), Momma & Izumi (2008 ▸).

## 6. Conclusions

Computational methods with exceptional accuracy are required for reliable polymorph prediction in crystal structure prediction (CSP) studies. However, state-of-the-art methods are associated with large computing costs and typically rely on lattice energies to determine relative stabilities of polymorphs. This approach neglects important thermal contributions to the stability of structures, which can change the relative energy ranking of polymorphs, as well as provide vital information as to how predicted phases may behave under different conditions. Free energies therefore provide a more accurate measure, but are generally neglected in CSP workflows as their calculation increases the computational cost further. Therefore, for accurate and efficient polymorph prediction alternative approaches must be considered.

Here we have used the FFLUX force field to study three phases of ice (I*h*, II and XV), calculating Gibbs free energies for the first time using quasi-harmonic lattice dynamics calculations. FFLUX uses Gaussian process regression (GPR) models to predict an intramolecular potential energy surface with quantum mechanical accuracy and multipole moments for electrostatic interactions. These models allow for flexible molecules where the multipole moments change with geometry on-the-fly during simulations, features that are not common in force fields typically used in CSP. While costly with typical CSP methods, we show that FFLUX can efficiently perform lattice dynamics calculations to obtain both Helmholtz and Gibbs free energies approximately 10^6^–10^7^ times faster than periodic plane-wave DFT calculations.

Optimization of the crystal structures showed that FFLUX was able to obtain lattice parameters comparable with those obtained from PBE+D3 at a significantly reduced computational cost, with all lattice parameters obtained within 5% of experimental values. Despite the success in reproducing the unit cells, the relative lattice energy ranking obtained with FFLUX did not follow chemical intuition, with ice II being predicted as less stable than the higher-pressure ice XV. Given the sub-kJ mol^−1^ accuracy of the presented GPR model, these errors were primarily attributed to the parametrized non-bonded potential, which is external and hence not part of the general architecture of FFLUX. However, due to the monomeric nature of the GPR model, the lack of intermolecular polarization effects is also likely to be contributing to the errors seen in these calculations.

Harmonic phonon calculations allowed the dynamic stability of the three ices to be assessed. While PBE+D3 found all three phases to be stable, FFLUX incorrectly identified ice II as unstable. Mapping the instability led to the identification of (to our knowledge) a new phase of ice, labelled here as ice II′. This phase was also found to be dynamically stable in PBE+D3 calculations, but less stable than ice II, in contrast to FFLUX predictions. The discovery of ice II′ is again likely due to the non-bonded potential used, which artificially stabilized II′ over ice II. The non-bonded potential also causes relative Helmholtz free energies to be largely overpredicted compared with PBE+D3 and experimental data, which is then problematic in the quasi-harmonic calculations. In these calculations, small free energy errors can lead to large deviations in the locations of phase transitions (Červinka & Beran, 2018 ▸). Here, the errors present themselves as FFLUX overpredicting the transition pressure between I*h* and XV.

While currently problematic, the issues with non-bonded potentials are fixable within the FFLUX methodology. This fix comes in the form of machine-learning models trained to predict dispersion and repulsion, making non-bonded potentials (and their time-consuming parametrization) redundant. We have already shown that it is possible to machine-learn dynamic electron correlation energies, enabling the prediction of dispersive interactions (McDonagh *et al.*, 2018 ▸), as well as incorporate intermolecular repulsion by training oligomeric models (Brown *et al.*, 2024 ▸). Currently these oligomeric models can only be used for systems of the size for which they are trained (*i.e.* a dimer model may only be used for a dimer simulation), but work is underway to make extension to larger systems possible. Once implemented, FFLUX simulations will be closer to quantum mechanics. Moreover, all information will be coming from GPR models trained on data coming from one consistent and general scheme (interacting quantum atoms), making our method future-proof.

## Supplementary Material

Process used to determine the Lennard-Jones parameters (S1); comparison of the time taken for FFLUX and PBE+D3 optimisations (S2.1); the accuracy and cost of the central unit cell extraction method (S2.2); comparison of the time taken for FFLUX and PBE+D3 single-point force calculations (S2.3); further assessment of the performance of the GPR model (S3); FFLUX and PBE+D3 phonon dispersion curves (S4.1); description of the mode-map process and details of the structure of phase II' (S4.2). DOI: 10.1107/S2053273324010921/pen5005sup1.pdf

## Figures and Tables

**Figure 3:**
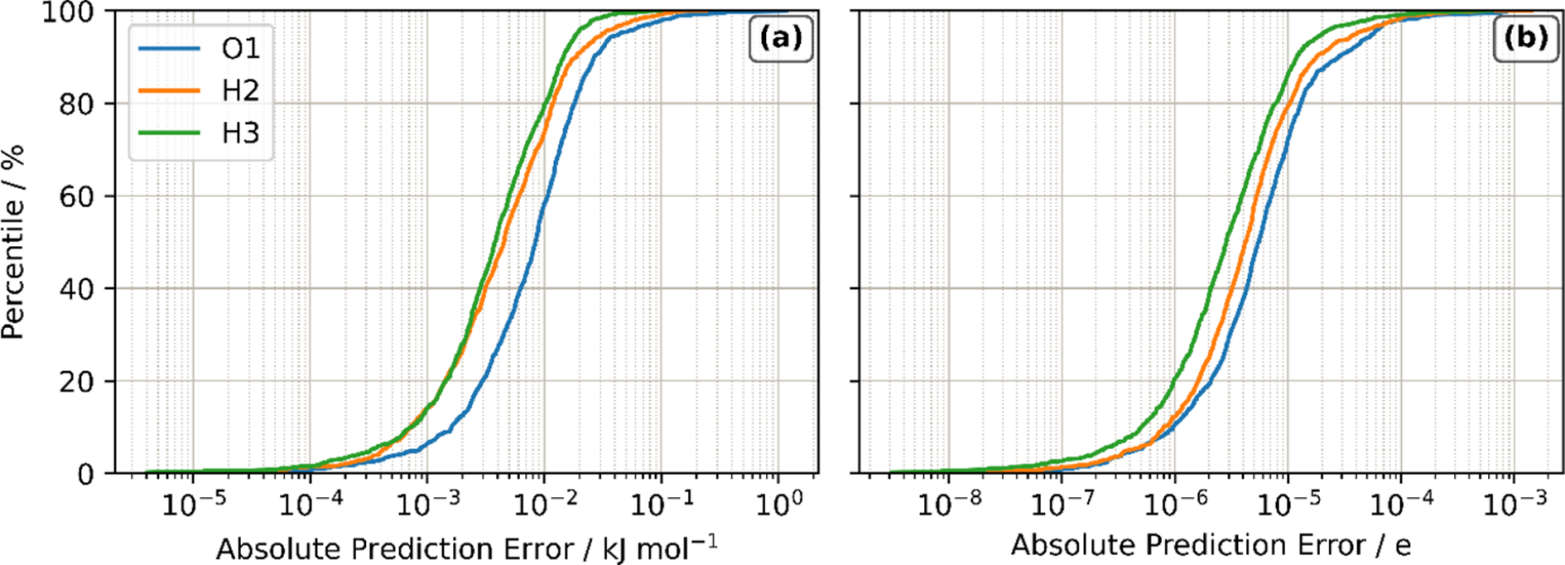
S-curves showing the absolute prediction error of (*a*) IQA energies and (*b*) charge of the atoms in the water monomer.

**Table 1:** ICSD codes for ice phases I*h* (Hamann, 1997 ▸), II (Kamb *et al.*, 1971 ▸) and XV (Salzmann *et al.*, 2009 ▸) studied in this work and supercell expansions used in FFLUX optimizations The space groups and the number of molecules in the supercells are also given.

Phase	ICSD code	Space group	Supercell	*N* molecules
I*h*	27837	*P*6_3_/*mmc*	4 × 4 × 4	768
II	23707	*R* 3	5 × 5 × 5	1500
XV	166447	*P* 1	4 × 4 × 5	800

**Table 2:** Summary of the technical parameters used for the geometry optimizations and phonon calculations on the studied ice polymorphs

	**k**-point sampling			
Phase	Optimization	Phonon	Supercell expansion	No. of supercell molecules
I*h*	3 × 3 × 2	2 × 2 × 1	2 × 2 × 2	96
II	2 × 2 × 2	1 × 1 × 1	2 × 2 × 2	96
XV	2 × 2 × 3	1 × 1 × 2	2 × 2 × 2	80

**Table 3:** Comparison of unit-cell parameters predicted by FFLUX and PBE+D3 with experiment

Phase	*a*, *b*, *c* (Å)	Δ%	α, β, γ (°)	Δ%	*V* (Å^3^)	Δ%
I*h*						
Experiment	7.82, 7.82, 7.36		90.0, 90.0, 120.0		389.78	
I*h* FFLUX	7.61, 7.63, 7.14	−2.6, −2.5, −2.9	89.8, 90.0, 120.2	−0.2, 0.0, 0.2	358.53	−8.0
I*h* PBE+D3	7.54, 7.54, 7.08	−3.6, −3.6, −3.8	90.0, 90.0, 120.0	0.0, 0.0, 0.0	348.18	−10.7
						
II						
Experiment	7.78, 7.78, 7.78		113.1, 113.1, 113.1		304.25	
II FFLUX	7.79, 7.79, 7.76	0.2, 0.2, −0.3	113.0, 112.8, 113.4	−0.1, −0.2, 0.3	304.64	0.1
II PBE+D3	7.52, 7.52, 7.52	−3.3, −3.3, −3.3	113.0, 113.0, 113.0	−0.1, −0.1, −0.1	277.30	−8.9
						
XV						
Experiment	6.23, 6.24, 5.79		90.1, 90.0, 89.9		225.32	
XV FFLUX	6.36, 6.29, 5.83	2.0, 0.7, 0.7	92.2, 91.8, 91.0	2.3, 2.0, 1.1	232.79	3.3
XV PBE+D3	6.08, 6.07, 5.67	−2.4, −2.7, −2.1	90.5, 89.6, 90.5	0.5, −0.4, 0.7	209.27	−7.1

**Table 4:** Lattice energies (in kJ mol^−1^) calculated using FFLUX and PBE+D3 Experimental energies extrapolated to zero temperature for phases I*h* and II (Whalley, 1984 ▸) are also given.

	FFLUX	Relative FFLUX	PBE+D3	Relative PBE+D3	Experiment	Relative experiment
I*h*	−59.6	0.0	−73.1	0.0	−58.9	0.0
II	−41.0	18.6	−70.6	2.5	−58.8	0.1
XV	−44.0	15.6	−68.0	5.1	–	–

## Data Availability

The data supporting the findings in this paper are available free of charge: ‘Data for: A Computationally Efficient Quasi-Harmonic Study of Ice Polymorphs Using the FFLUX Force Field’ at DOI:10.17632/8r3cz73k3v.1.

## References

[bb1] Abascal, J. L. F., Sanz, E., García Fernández, R. & Vega, C. (2005). *J. Chem. Phys.* **122**, 234511. 10.1063/1.1931662 16008466

[bb2] Bader, R. F. W. (1985). *Acc. Chem. Res.* **18**, 9–15.

[bb3] Bauer, J., Spanton, S., Henry, R., Quick, J., Dziki, W., Porter, W. & Morris, J. (2001). *Pharm. Res.* **18**, 859–866. 10.1023/a:1011052932607 11474792

[bb4] Beran, G. J. O. (2023). *Chem. Sci.* **14**, 13290–13312. 10.1039/d3sc03903j PMC10685338 38033897

[bb5] Bernal, J. D. & Fowler, R. H. (1933). *J. Chem. Phys.* **1**, 515–548.

[bb6] Birch, F. (1947). *Phys. Rev.* **71**, 809–824.

[bb7] Blanco, M. A., Martín Pendás, A. & Francisco, E. (2005). *J. Chem. Theory Comput.* **1**, 1096–1109. 10.1021/ct0501093 26631653

[bb8] Blöchl, P. E. (1994). *Phys. Rev. B*, **50**, 17953–17979. 10.1103/physrevb.50.17953 9976227

[bb9] Bore, S. L. & Paesani, F. (2023). *Nat. Commun.* **14**, 3349. 10.1038/s41467-023-38855-1 PMC10250386 37291095

[bb10] Brown, M. L., Isamura, B. K., Skelton, J. M. & Popelier, P. L. A. (2024). *J. Chem. Theory Comput.* **20**, 5994–6008. 10.1021/acs.jctc.4c00402 PMC11270819 38981081

[bb11] Brown, M. L., Skelton, J. M. & Popelier, P. L. A. (2023*a*). *J. Chem. Theory Comput.* **19**, 7946–7959. 10.1021/acs.jctc.3c00578 PMC10653110 37847867

[bb12] Brown, M. L., Skelton, J. M. & Popelier, P. L. A. (2023*b*). *J. Phys. Chem. A*, **127**, 1702–1714. 10.1021/acs.jpca.2c06566 PMC9969515 36756842

[bb13] Bučar, D. K., Lancaster, R. W. & Bernstein, J. (2015). *Angew. Chem. Int. Ed.* **54**, 6972–6993. 10.1002/anie.201410356 PMC4479028 26031248

[bb14] Burn, M. J. & Popelier, P. L. A. (2022). *Mater. Adv.* **3**, 8729–8739.

[bb15] Burn, M. J. & Popelier, P. L. A. (2023). *Digit. Discov.* **2**, 152–164.

[bb16] Červinka, C. & Beran, G. J. O. (2018). *Chem. Sci.* **9**, 4622–4629. 10.1039/c8sc01237g PMC5969506 29899955

[bb17] Clough, S. A., Beers, Y., Klein, G. P. & Rothman, L. S. (1973). *J. Chem. Phys.* **59**, 2254–2259.

[bb18] Day, G. M., Cooper, T. G., Cruz-Cabeza, A. J., Hejczyk, K. E., Ammon, H. L., Boerrigter, S. X. M., Tan, J. S., Della Valle, R. G., Venuti, E., Jose, J., Gadre, S. R., Desiraju, G. R., Thakur, T. S., van Eijck, B. P., Facelli, J. C., Bazterra, V. E., Ferraro, M. B., Hofmann, D. W. M., Neumann, M. A., Leusen, F. J. J., Kendrick, J., Price, S. L., Misquitta, A. J., Karamertzanis, P. G., Welch, G. W. A., Scheraga, H. A., Arnautova, Y. A., Schmidt, M. U., van de Streek, J., Wolf, A. K. & Schweizer, B. (2009). *Acta Cryst.* B**65**, 107–125. 10.1107/S0108768109004066 19299868

[bb19] Di Pasquale, N., Bane, M., Davie, S. J. & Popelier, P. L. A. (2016). *J. Comput. Chem.* **37**, 2606–2616. 10.1002/jcc.24486 27649926

[bb20] Dykstra, C. E. (1988). *Acc. Chem. Res.* **21**, 356–361.

[bb21] Essmann, U., Perera, L., Berkowitz, M. L., Darden, T., Lee, H. & Pedersen, L. G. (1995). *J. Chem. Phys.* **103**, 8577–8593.

[bb22] Fornaro, T., Carnimeo, I. & Biczysko, M. (2015). *J. Phys. Chem. A*, **119**, 5313–5326. 10.1021/jp510101y 25474755

[bb23] Freedman, D. & Diaconis, P. (1981). *Z. Wahrscheinlichkeitstheorie Verwandte Gebiete*, **57**, 453–476.

[bb24] Frisch, M. J., Trucks, G. W., Schlegel, H. B., Scuseria, G. E., Robb, M. A., Cheeseman, J. R., Scalmani, G., Barone, V., Mennucci, B. & Petersson, G. A. (2010). *GAUSSIAN09*. Gaussian Inc., Wallingford, CT, USA.

[bb90] Frisch, M. J. *et al.* (2016). *GAUSSIAN16*. Gaussian Inc., Wallingford, CT, USA.

[bb91] Fu, X., Wu, Z., Wang, W., Xie, T., Keten, S., Gomez-Bombarelli, R. & Jaakkola, T. (2023). *Trans. Mach. Learn. Res*. https://openreview.net/forum?id=A8pqQipwkt.

[bb25] Gasser, T. M., Thoeny, A. V., Fortes, A. D. & Loerting, T. (2021). *Nat. Commun.* **12**, 1128. 10.1038/s41467-021-21161-z PMC7892819 33602946

[bb26] Grimme, S., Antony, J., Ehrlich, S. & Krieg, H. (2010). *J. Chem. Phys.* **132**, 154104–154122. 10.1063/1.3382344 20423165

[bb27] Hamann, D. R. (1997). *Phys. Rev. B*, **55**, R10157–R10160.

[bb92] Hédin, F., El Hage, K. & Meuwly, M. (2016). *J. Chem. Inf. Model.* **56**, 1479–1489. 10.1021/acs.jcim.6b00280 27438992

[bb28] Hellenbrandt, M. (2004). *Crystallogr. Rev.* **10**, 17–22.

[bb29] Hellman, O., Steneteg, P., Abrikosov, I. A. & Simak, S. I. (2013). *Phys. Rev. B*, **87**, 104111.

[bb30] Hunnisett, L. M., Francia, N., Nyman, J., Abraham, N. S., Aitipamula, S., Alkhidir, T., Almehairbi, M., Anelli, A., Anstine, D. M., Anthony, J. E., Arnold, J. E., Bahrami, F., Bellucci, M. A., Beran, G. J. O., Bhardwaj, R. M., Bianco, R., Bis, J. A., Boese, A. D., Bramley, J., Braun, D. E., Butler, P. W. V., Cadden, J., Carino, S., Cervinka, C., Chan, E. J., Chang, C., Clarke, S. M., Coles, S. J., Cook, C. J., Cooper, R. I., Darden, T., Day, G. M., Deng, W., Dietrich, H., DiPasquale, A., Dhokale, B., van Eijck, B. P., Elsegood, M. R. J., Firaha, D., Fu, W., Fukuzawa, K., Galanakis, N., Goto, H., Greenwell, C., Guo, R., Harter, J., Helfferich, J., Hoja, J., Hone, J., Hong, R., Husak, M., Ikabata, Y., Isayev, O., Ishaque, O., Jain, V., Jin, Y., Jing, A., Johnson, E. R., Jones, I., Jose, K. V. J., Kabova, E. A., Keates, A., Kelly, P. F., Klimes, J., Kostkova, V., Li, H., Lin, X., List, A., Liu, C., Liu, Y. M., Liu, Z., Loncaric, I., Lubach, J. W., Ludik, J., Marom, N., Matsui, H., Mattei, A., Mayo, R. A., Melkumov, J. W., Mladineo, B., Mohamed, S., Momenzadeh Abardeh, Z., Muddana, H. S., Nakayama, N., Nayal, K. S., Neumann, M. A., Nikhar, R., Obata, S., O’Connor, D., Oganov, A. R., Okuwaki, K., Otero-de-la-Roza, A., Parkin, S., Parunov, A., Podeszwa, R., Price, A. J. A., Price, L. S., Price, S. L., Probert, M. R., Pulido, A., Ramteke, G. R., Rehman, A. U., Reutzel-Edens, S. M., Rogal, J., Ross, M. J., Rumson, A. F., Sadiq, G., Saeed, Z. M., Salimi, A., Sasikumar, K., Sekharan, S., Shankland, K., Shi, B., Shi, X., Shinohara, K., Skillman, A. G., Song, H., Strasser, N., van de Streek, J., Sugden, I. J., Sun, G., Szalewicz, K., Tan, L., Tang, K., Tarczynski, F., Taylor, C. R., Tkatchenko, A., Tom, R., Tous, P., Tuckerman, M. E., Unzueta, P. A., Utsumi, Y., Vogt-Maranto, L., Weatherston, J., Wilkinson, L. J., Willacy, R. D., Wojtas, L., Woollam, G. R., Yang, Y., Yang, Z., Yonemochi, E., Yue, X., Zeng, Q., Zhou, T., Zhou, Y., Zubatyuk, R. & Cole, J. C. (2024). *Acta Cryst.* B**80**, https://doi.org/10.1107/S2052520624008679.

[bb31] Isamura, B. K. & Popelier, P. L. A. (2023*a*). *AIP Adv.* **13**, 095202.

[bb32] Isamura, B. K. & Popelier, P. L. A. (2023*b*). *Artif. Intell. Chem.* **1**, 100021.

[bb33] Jorgensen, W. L., Chandrasekhar, J., Madura, J. D., Impey, R. W. & Klein, M. L. (1983). *J. Chem. Phys.* **79**, 926–935.

[bb34] Kamb, B., Hamilton, W. C., LaPlaca, S. J. & Prakash, A. (1971). *J. Chem. Phys.* **55**, 1934–1945.

[bb35] Keith, T. A. (2019). *AIMAll, AIMStudio*. TK Gristmill Software, Overland Park, Kansas, USA.

[bb36] Konovalov, A., Symons, B. C. B. & Popelier, P. L. A. (2021). *J. Comput. Chem.* **42**, 107–116. 10.1002/jcc.26438 33107993

[bb37] Kresse, G. & Hafner, J. (1993). *Phys. Rev. B*, **47**, 558–561. 10.1103/physrevb.47.558 10004490

[bb38] Kresse, G. & Joubert, D. (1999). *Phys. Rev. B*, **59**, 1758–1775.

[bb39] Manchev, Y. T. & Burn, M. J. (2024). *ichor: Computational Chemistry Data Management Library for Machine Learning Force Field Development.* Version 4.0.3. University of Manchester, UK. 10.1002/jcc.27477 39215569

[bb40] McDonagh, J. L., Silva, A. F., Vincent, M. A. & Popelier, P. L. A. (2018). *J. Chem. Theory Comput.* **14**, 216–224. 10.1021/acs.jctc.7b01157 29211469

[bb41] Mills, M. J. L. & Popelier, P. L. A. (2014). *J. Chem. Theory Comput.* **10**, 3840–3856. 10.1021/ct500565g 26588529

[bb93] Momma, K. & Izumi, F. (2008). *J. Appl. Cryst.* **41**, 653–658.

[bb42] Nyman, J. & Day, G. M. (2015). *CrystEngComm*, **17**, 5154–5165.

[bb43] Pallikara, I., Kayastha, P., Skelton, J. M. & Whalley, L. D. (2022). *Electron. Struct.* **4**, 033002.

[bb44] Pallikara, I. & Skelton, J. M. (2021). *Phys. Chem. Chem. Phys.* **23**, 19219–19236. 10.1039/d1cp02597j 34524313

[bb45] Perdew, J. P., Burke, K. & Ernzerhof, M. (1996). *Phys. Rev. Lett.* **77**, 3865–3868. 10.1103/PhysRevLett.77.3865 10062328

[bb46] Popelier, P. L. A. (2015). *Int. J. Quantum Chem.* **115**, 1005–1011.

[bb47] Popelier, P. L. A. (2016). *Challenges and Advances in Computational Chemistry and Physics dedicated to ‘Applications of Topological Methods in Molecular Chemistry’*, edited by R. Chauvin, C. Lepetit, E. Alikhani & B. Silvi, pp. 23–52. Switzerland: Springer.

[bb48] Prask, H. J., Trevino, S. F., Gault, J. D. & Logan, K. W. (1972). *J. Chem. Phys.* **56**, 3217–3225.

[bb49] Price, S. L., Leslie, M., Welch, G. W. A., Habgood, M., Price, L. S., Karamertzanis, P. G. & Day, G. M. (2010). *Phys. Chem. Chem. Phys.* **12**, 8478–8490. 10.1039/c004164e 20607186

[bb50] Rahim, W., Skelton, J. M., Savory, C. N., Evans, I. R., Evans, J. S. O., Walsh, A. & Scanlon, D. O. (2020). *Chem. Sci.* **11**, 7904–7909. 10.1039/d0sc02995e PMC8617592 34909139

[bb51] Ramírez, R., Neuerburg, N. & Herrero, C. P. (2012). *J. Chem. Phys.* **137**, 044502. 10.1063/1.4737862 22852626

[bb52] Ramírez, R., Neuerburg, N. & Herrero, C. P. (2013). *J. Chem. Phys.* **139**, 084503. 10.1063/1.4818875 24007014

[bb53] Rasmussen, C. E. & Williams, C. K. I. (2006). *Gaussian Processes for Machine Learning.* Cambridge, USA: The MIT Press.

[bb54] Reilly, A. M., Cooper, R. I., Adjiman, C. S., Bhattacharya, S., Boese, A. D., Brandenburg, J. G., Bygrave, P. J., Bylsma, R., Campbell, J. E., Car, R., Case, D. H., Chadha, R., Cole, J. C., Cosburn, K., Cuppen, H. M., Curtis, F., Day, G. M., DiStasio, R. A. Jr, Dzyabchenko, A., van Eijck, B. P., Elking, D. M., van den Ende, J. A., Facelli, J. C., Ferraro, M. B., Fusti-Molnar, L., Gatsiou, C.-A., Gee, T. S., de Gelder, R., Ghiringhelli, L. M., Goto, H., Grimme, S., Guo, R., Hofmann, D. W. M., Hoja, J., Hylton, R. K., Iuzzolino, L., Jankiewicz, W., de Jong, D. T., Kendrick, J., de Klerk, N. J. J., Ko, H.-Y., Kuleshova, L. N., Li, X., Lohani, S., Leusen, F. J. J., Lund, A. M., Lv, J., Ma, Y., Marom, N., Masunov, A. E., McCabe, P., McMahon, D. P., Meekes, H., Metz, M. P., Misquitta, A. J., Mohamed, S., Monserrat, B., Needs, R. J., Neumann, M. A., Nyman, J., Obata, S., Oberhofer, H., Oganov, A. R., Orendt, A. M., Pagola, G. I., Pantelides, C. C., Pickard, C. J., Podeszwa, R., Price, L. S., Price, S. L., Pulido, A., Read, M. G., Reuter, K., Schneider, E., Schober, C., Shields, G. P., Singh, P., Sugden, I. J., Szalewicz, K., Taylor, C. R., Tkatchenko, A., Tuckerman, M. E., Vacarro, F., Vasileiadis, M., Vazquez-Mayagoitia, A., Vogt, L., Wang, Y., Watson, R. E., de Wijs, G. A., Yang, J., Zhu, Q. & Groom, C. R. (2016). *Acta Cryst.* B**72**, 439–459.

[bb55] Salzmann, C. G., Radaelli, P. G., Hallbrucker, A., Mayer, E. & Finney, J. L. (2006). *Science*, **311**, 1758–1761. 10.1126/science.1123896 16556840

[bb56] Salzmann, C. G., Radaelli, P. G., Mayer, E. & Finney, J. L. (2009). *Phys. Rev. Lett.* **103**, 105701. 10.1103/PhysRevLett.103.105701 19792330

[bb57] Schrodinger (2015). *The *pyMOL* Molecular Graphics System*, Version 2.5. Schrodinger, LLC.

[bb58] Sinha, P., Boesch, S. E., Gu, C., Wheeler, R. A. & Wilson, A. K. (2004). *J. Phys. Chem. A*, **108**, 9213–9217.

[bb59] Skelton, J. M., Burton, L. A., Parker, S. C., Walsh, A., Kim, C.-E., Soon, A., Buckeridge, J., Sokol, A. A., Catlow, C. R. A., Togo, A. & Tanaka, I. (2016). *Phys. Rev. Lett.* **117**, 075502. 10.1103/PhysRevLett.117.075502 27563974

[bb60] Symons, B. C. B., Bane, M. K. & Popelier, P. L. A. (2021). *J. Chem. Theory Comput.* **17**, 7043–7055. 10.1021/acs.jctc.1c00595 PMC8582247 34617748

[bb61] Symons, B. C. B. & Popelier, P. L. A. (2022). *J. Chem. Theory Comput.* **18**, 5577–5588. 10.1021/acs.jctc.2c00311 PMC9476653 35939826

[bb62] Todorov, I. T. & Smith, W. (2018). *The DL_POLY_4 User Manual*. CCLRC Daresbury Laboratory, Warrington, UK.

[bb63] Togo, A. & Tanaka, I. (2015). *Scr. Mater.* **108**, 1–5.

[bb64] Vega, C., Sanz, E., Abascal, J. L. F. & Noya, E. G. (2008). *J. Phys. Condens. Matter*, **20**, 153101.

[bb65] Wang, J., Wolf, R. M., Caldwell, J. W., Kollman, P. A. & Case, D. A. (2004). *J. Comput. Chem.* **25**, 1157–1174. 10.1002/jcc.20035 15116359

[bb66] Whalley, E. (1984). *J. Chem. Phys.* **81**, 4087–4092.

