# Supplementary material for: A computationally efficient quasi-harmonic study of ice polymorphs using the FFLUX force field
Source: Acta Crystallogr A Found Adv. 2025 Jan 1;81(Pt 1):36–48. doi: 10.1107/S2053273324010921 (PMC11694218; doi:10.1107/S2053273324010921)
Supplement: Supplementary file 1 [file a-81-00036-sup1.pdf]

Acta Cryst A
FOUNDATIONS
ADVANCES

Volume 81 (2025)

Supporting information for article:

A computationally efficient quasi-harmonic study of ice polymorphs using the FFLUX force field

Alexandra Pák, Matthew L. Brown and Paul Popelier

Supporting Information

A Computationally Efficient Quasi-Harmonic Study of Ice Polymorphs Using the FFLUX Force Field

Alexandra Pák, Matthew L. Brown and Paul L. A. Popelier

Contents

S1. Non-bonded parameters S2

S2. Efficiency of calculations S4

S2.1. Evaluation of the computational cost of crystal structure optimisations S4

S2.2. Cost and accuracy of central unit cell extraction S7

S2.3. Evaluation of the computational cost of lattice dynamics calculations S8

S3. GPR model performance S10

S4. Harmonic approximation S26

S4.1. Phonon dispersion curves S26

S4.2. Phase II' S27

References S32

S1. Non-bonded parameters

The Gaussian process regression (GPR) models used in the FFLUX simulations in this work are monomeric, meaning that they have been trained on monomeric calculations. The monomeric nature of the training data therefore means that the models cannot predict intermolecular dispersive and repulsive interactions. Although this is possible within the FFLUX workflow (McDonagh *et al.*, 2018, Brown *et al.*, 2024) through the use of oligomeric models, this approach has not been applied here. To account for these interactions here, a 12-6 Lennard-Jones (LJ) potential was used:

$$U_{ij}(r) = \frac{A_{ij}}{r^{12}} - \frac{B_{ij}}{r^6} \tag{S1}$$

where $A_{ij} = 4\varepsilon\sigma^{12}$ and $B_{ij} = 4\varepsilon\sigma^6$, ε is the potential well depth and σ is the separation at which the potential energy is zero. As suggested by previous work (Brown *et al.*, 2023), in order to accurately model different polymorphs each phase would require a different set of nonbonded parameters. Given the large variance in the molecular environments of the different phases, it is expected that intermolecular interactions also significantly differ and therefore the same parameters will not be suitable for all phases.

Initially, the Lennard-Jones parameters from previous work on liquid water (Symons & Popelier, 2022) were used for all phases, however, these were found to be suitable only for phase XV. Therefore, to find the parameters for phases Ih and II, the TIP4P/Ice Lennard-Jones parameters (Abascal *et al.*, 2005) were used as a starting point. The A and B values were scaled separately by different factors n and m to obtain new parameters A^* and B^*:

$$A_{ij}^* = nA_{ij} \tag{S2}$$

$$B_{ij}^* = mB_{ij} \tag{S3}$$

where n and m were varied between 0.5 to 2.0 in steps of 0.025.

A series of optimisations (described in Section 3.2 of the main text) were performed with the varied LJ parameters. Subsequently, a score was calculated for the parameter sets so that we could determine the appropriate values to be used for each phase. The score took into account the cell lengths, angles and the densities of the optimised structures compared to the experimental values in a weighted sum of square differences, as performed by Meuwly *et al.* (Hédin *et al.*, 2016) and in previous work (Brown *et al.*, 2023):

$$S = \sum_p w_p\left(calc_p - exp_p\right)^2 \tag{S4}$$

where exp_p is the experimental property and $calc_p$ the calculated one. Weighting factors, w_p, were given to each property based on the size of the error from experimental values. The parameter set that obtained the

structure with the lowest score S was taken as the best parameter set. The selected parameters for each phase are shown in Table S1.

Table S1 Lennard-Jones parameters of the FFLUX-optimised ice structures for oxygen.

Phase	A (kJ Å^{12} mol^{-1})	B (kJ Å^{6} mol^{-1})
I*h*	2,742,342.550	4,675.858
II	2,333,144.840	1,779.246
XV	3,142,647.540	3,096.992

Weights used for the cell lengths, cell angles and densities for phases I*h* and II were 10, 10 and 100 respectively. Larger weights were given to the densities as these generally differed less from the experimental values compared to the cell parameters. Densities of the experimental structures used to calculate the scores were 0.92 and 1.18 g cm^{-3} respectively.

S2. Efficiency of calculations

S2.1. Evaluation of the computational cost crystal structure optimisations

Supercell optimisations using FFLUX used 4 cores and PBE+D3 used 80 cores of two compute nodes, each consisting of two Intel "Cascade Lake" Xeon Gold 6230 chips. The number of atoms contained in the supercells of the ices used by FFLUX and PBE+D3 are shown in Table S2.

Table S2 Time taken for FFLUX and PBE+D3 optimisations for the ice phases studied using the VASP code. Number of cores N_{Proc} and number of atoms are also given.

Phase	N_{Proc}	N_{atoms}	Average Time / s	CPU Time / core hr
Ih PBE+D3	80	36	471.54	10.48
Ih FFLUX	4	2304	9690.64	35.08
II PBE+D3	80	36	230.19	6.12
II FFLUX	4	4500	21868.41	68.46
XV PBE+D3	80	30	344.57	7.66
XV FFLUX	1	2400	13284.76	40.50

We can estimate the time taken for a FFLUX-sized supercell of each phase and compare the costs of optimisations assuming VASP scales between N^2 and N^3:

Phase I*h*:

<u>Scaling of system size:</u>

$$\frac{2\,304 \text{ atoms}}{36 \text{ atoms}} = 64$$

<u>Converting to core hours:</u>

VASP time (core hr):

$$\frac{471.54 \text{ s}}{3\,600 \text{ s hr}^{-1}} \times 80 \text{ cores} = 10.48 \text{ core hr}$$

FFLUX time (core hr):

$$\frac{9\ 690.64\ \text{s}}{3\ 600\ \text{s hr}^{-1}} \times 4\ \text{cores} = 35.08\ \text{core hr}$$

Assuming N^2 scaling:

$$10.48\ \text{core hr} \times 64^2 = 42\ 926\ \text{core hr}$$

$$\frac{42\ 926\ \text{core hr}}{35.08\ \text{core hr}} = 1\ 224\ \text{times slower than FFLUX}$$

Assuming N^3 scaling:

$$10.48\ \text{core hr} \times 64^3 = 2\ 747\ 269.12\ \text{core hr}$$

$$\frac{2\ 747\ 269\ \text{core hr}}{35.08\ \text{core hr}} = 78\ 309\ \text{times slower than FFLUX}$$

Phase II:

Scaling of system size:

$$\frac{4\ 500\ \text{atoms}}{36\ \text{atoms}} = 125$$

Converting to core hours:

VASP time (core hr):

$$\frac{230.19\ \text{s}}{3\ 600\ \text{s hr}^{-1}} \times 80\ \text{cores} = 6.12\ \text{core hr}$$

FFLUX time (core hr):

$$\frac{21\ 868.41\ \text{s}}{3\ 600\ \text{s hr}^{-1}} \times 4\ \text{cores} = 68.46\ \text{core hr}$$

Assuming N^2 scaling:

$$6.12\ \text{core hr} \times 125^2 = 95\ 625\ \text{core hr}$$

$$\frac{95\ 625\ \text{core hr}}{68.46\ \text{core hr}} = 1\ 397\ \text{times slower than FFLUX}$$

Assuming N^3 scaling:

$$6.12\ \text{core hr} \times 125^3 = 11\ 953\ 125\ \text{core hr}$$

$$\frac{11\ 953\ 125\ \text{core hr}}{68.46\ \text{core hr}} = 174\ 604\ \text{times slower than FFLUX}$$

Phase XV:

Scaling of system size:

$$\frac{2\ 400 \text{ atoms}}{30 \text{ atoms}} = 80$$

Converting to core hours:

VASP time (core hr):

$$\frac{344.57 \text{ s}}{3\ 600 \text{ s hr}^{-1}} \times 80 \text{ cores} = 7.66 \text{ core hr}$$

FFLUX time (core hr):

$$\frac{13\ 284.76 \text{ s}}{3\ 600 \text{ s hr}^{-1}} \times 4 \text{ cores} = 40.50 \text{ core hr}$$

Assuming N^2 scaling:

$$7.66 \text{ core hr} \times 80^2 = 49\ 024 \text{ core hr}$$

$$\frac{49\ 024 \text{ core hr}}{40.50 \text{ core hr}} = 1\ 210 \text{ times slower than FFLUX}$$

Assuming N^3 scaling:

$$7.66 \text{ core hr} \times 80^3 = 3\ 921\ 920 \text{ core hr}$$

$$\frac{3\ 921\ 920 \text{ core hr}}{40.50 \text{ core hr}} = 96\ 838 \text{ times slower than FFLUX}$$

Using FFLUX enables us to simulate larger supercells, while performing 10^3 – 10^5 faster than VASP.

S2.2. Cost and Accuracy of central unit cell extraction

In order to obtain the force constants using the finite-displacement method implemented in Phonopy (Togo & Tanaka, 2015), up to six displacements are generated per atom in the structure provided to Phonopy, depending on the calculated symmetry. The FFLUX-optimised supercell of ice I*h* contained 2,304 atoms, compared to its unit cell that contained 36 atoms and at the default symmetry tolerance Phonopy assumes these structures to have P1 symmetry. This low symmetry group means that the maximum number of displaced structures are generated, with 13,824 and 216 structures for the supercell and the central unit cell, respectively. The total computing time required for the 13,824 single-point force calculations of the supercell was 43,662 s, while the 216 displaced structures of the unit cell required only 858 s. The extraction of the central unit cell is therefore approximately 50 times faster. After generating the displaced structures of the extracted unit cell, we can then expand it to its original size in Phonopy.

The accuracy of using the extracted central unit cell was tested by plotting the phonon density of states of both the supercell and central unit cell and calculating an RMSE between them. Figure S1 shows these DoS curves overlaid for ice I*h* calculated using FFLUX.

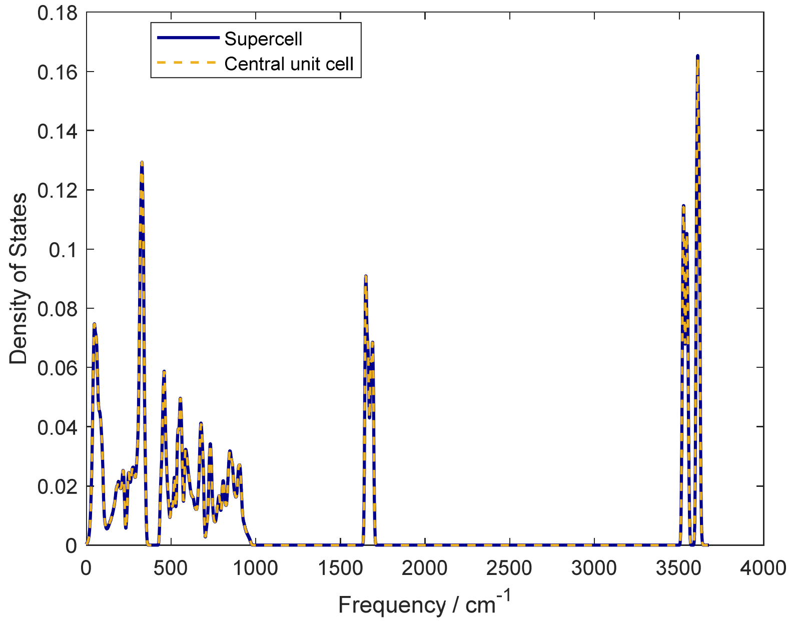

Figure S1 Phonon density of states of the ice Ih supercell (blue line) and central unit cell (orange dash).

The two plots can be seen to overlap almost perfectly, and the RMSE of the two was calculated to be 3.06×10^{-4}, meaning the difference is negligible and the extraction method can be reliably used in phonon calculations. The success of this method has demonstrated decreased computing time without any loss of accuracy, allowing for more efficient crystal structure prediction (CSP) in the future.

S2.3. Evaluation of the computational cost of lattice dynamics calculations

To avoid an increase in the cost of already expensive methods, many crystal structure prediction studies neglect free energy calculations. In the finite-displacement method, a series of structures are created where each atom is displaced from its equilibrium position in the optimised structure. The resulting atomic forces are then calculated using single-point calculations. The number of required displacements depends on symmetry; a higher symmetry structure will require fewer displacements.

For the FFLUX optimised supercells, 216 force calculations were required for ices Ih and II, and 180 for ice XV. Table S3 shows the average time of these calculations, given in seconds and core hours. Calculations using VASP used significantly smaller supercells than FFLUX. The number of atoms in each supercell is also shown. We can then estimate the time taken for a FFLUX-sized supercell and compare the costs for HA assuming VASP scales between N^2 and N^3.

Table S3 Average time taken for FFLUX and PBE+D3 single-point force calculations for the ice phases studied using the VASP code. Number of cores N_{Proc} and number of atoms are also given.

Phase	N_{Proc}	SC Atoms	Average Time / s	CPU Time/ core hr
Ih PBE+D3	40	288	7077.36	78.64
Ih FFLUX	1	2304	3.74	1.04×10^{-3}
II PBE+D3	40	288	615.54	6.84
II FFLUX	1	4500	7.99	2.22×10^{-3}
XV PBE+D3	40	240	1327.67	14.75
XV FFLUX	1	2400	3.36	9.32×10^{-4}

Computing times of VASP and FFLUX optimisations are compared only for phases II and XV, as VASP calculations for phase Ih were likely not ran with the most optimal settings, leading to increased computing times.

Phase II:

Scaling of system size:

$$\frac{4\ 500 \text{ atoms}}{288 \text{ atoms}} = 15.63$$

Converting to core hours:

$$\text{VASP time (core hr)} = \frac{615.54 \text{ s}}{3\ 600 \text{ s hr}^{-1}} \times 40 \text{ cores} \quad = 6.84 \text{ core hr}$$

$$\text{FFLUX time (core hr)} = \frac{7.99 \text{ s}}{3\ 600 \text{ s hr}^{-1}} \times 1 \text{ core} = 2.22 \times 10^{-3} \text{ core hr}$$

Assuming N^2 scaling:

$$6.84 \text{ core hr} \times 15.63^2 = 1\ 669.92 \text{ core hr}$$

$$\frac{1\ 669.92 \text{ core hr}}{2.22 \times 10^{-3} \text{ core hr}} = 759\ 055 \text{ times slower than FFLUX}$$

Assuming N^3 scaling:

$$6.84 \text{ core hr} \times 15.63^3 = 26\ 092.53 \text{ core hr}$$

$$\frac{26\ 092.53 \text{ core hr}}{2.22 \times 10^{-3} \text{ core hr}} = 11\ 860\ 241 \text{ times slower than FFLUX}$$

Phase XV:

Scaling of system size:

$$\frac{2\ 400 \text{ atoms}}{240 \text{ atoms}} = 10$$

Converting to core hours:

$$\text{VASP time (core hr)} = \frac{1\ 327.67 \text{ s}}{3\ 600 \text{ s hr}^{-1}} \times 40 \text{ cores} \quad = 14.75 \text{ core hr}$$

$$\text{FFLUX time (core hr)} = \frac{3.36 \text{ s}}{3\ 600 \text{ s hr}^{-1}} \times 1 \text{ core} = 9.34 \times 10^{-4}$$

Assuming N^2 scaling:

$$14.75 \text{ core hr} \times 10^2 = 1\ 475.20 \text{ core hr}$$

$$\frac{1\,475.20 \text{ core hr}}{9.32 \times 10^{-4} \text{ core hr}} = 1\,582\,934 \text{ times slower than FFLUX}$$

Assuming N^3 scaling:

$$14.75 \text{ core hr} \times 10^3 = 14\,750 \text{ core hr}$$

$$\frac{14\,750 \text{ core hr}}{9.32 \times 10^{-4} \text{ core hr}} = 15\,829\,345 \text{ times slower than FFLUX}$$

S3. GPR model performance

The predictive ability can be assessed by plotting cumulative distributions of the absolute prediction error over the external validation set, called S-curves. The predicted properties of all points in the set are compared to the true values, organised in ascending order then plotted against percentile. S-curves for the IQA energy and charge are provided in Figure 3 of the main text while here we provide S-curves for the remaining components multipole moments in Figures S2-S25.

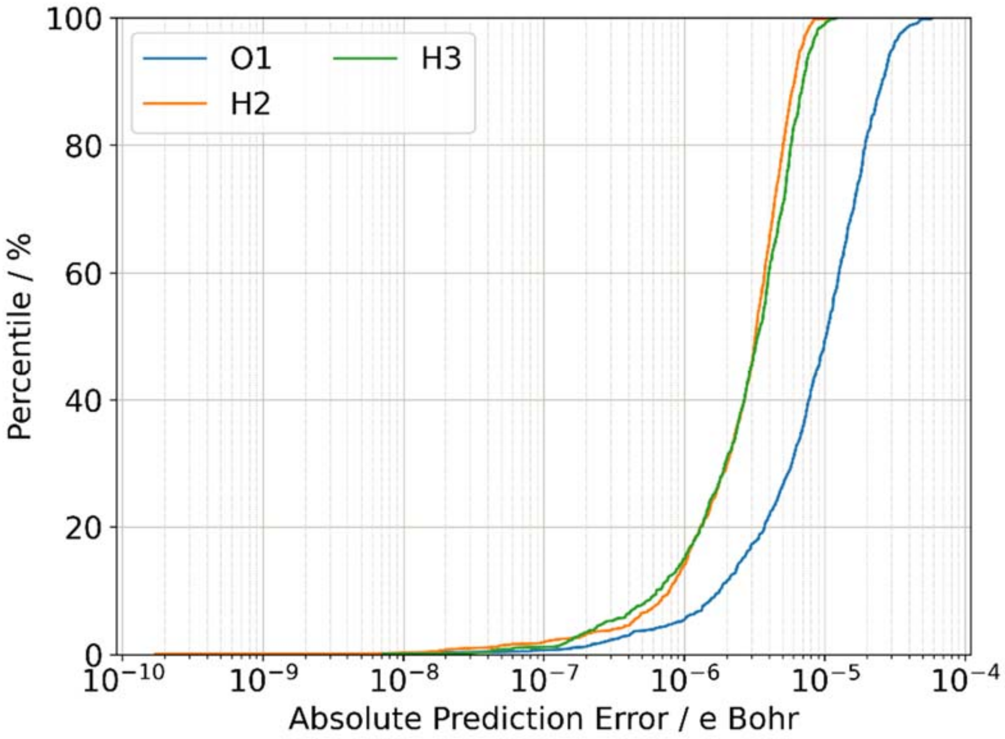

Figure S2 S-curves showing the absolute prediction error in the Q10 component of the atomic dipole moment of the water monomer model.

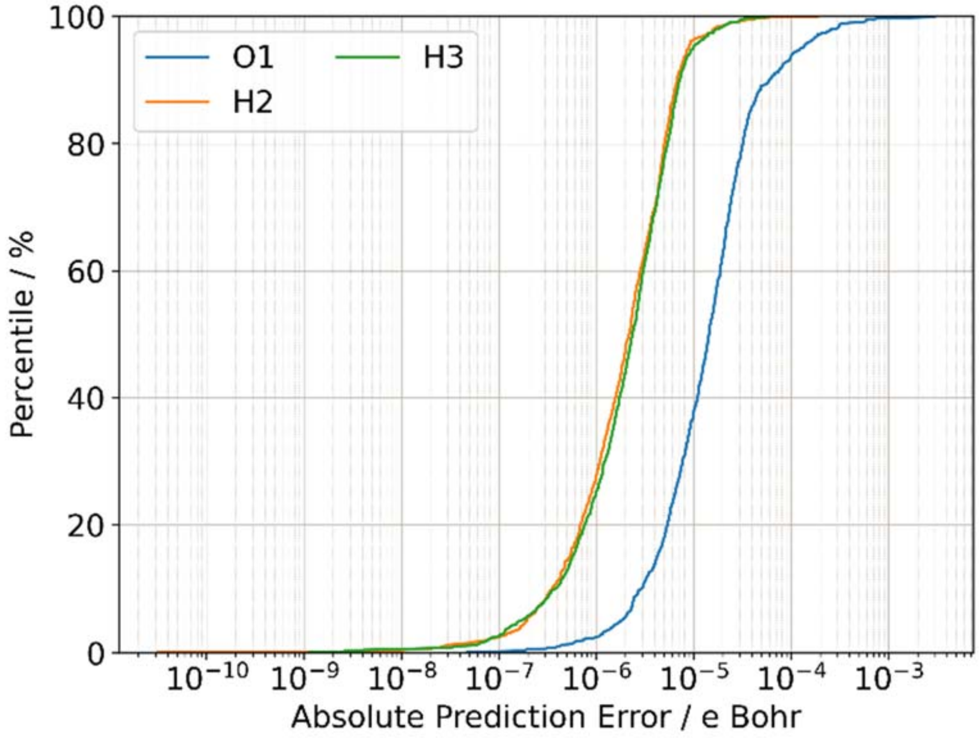

Figure S3 S-curves showing the absolute prediction error in the Q11c component of the atomic dipole moment of the water monomer model.

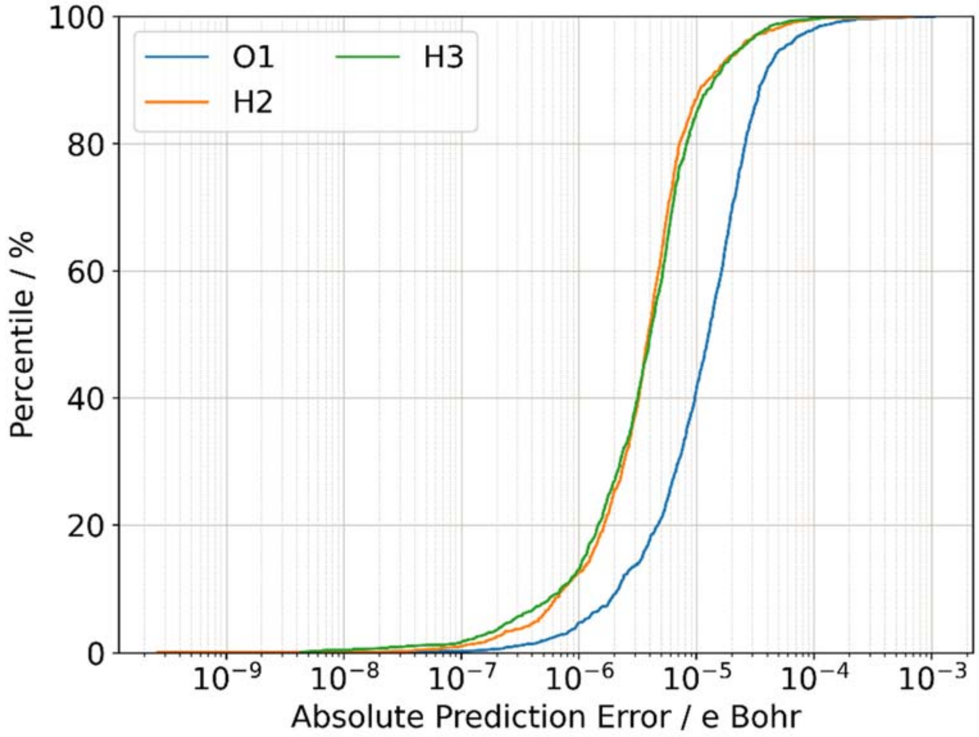

Figure S4 S-curves showing the absolute prediction error in the Q11s component of the atomic dipole moment of the water monomer model.

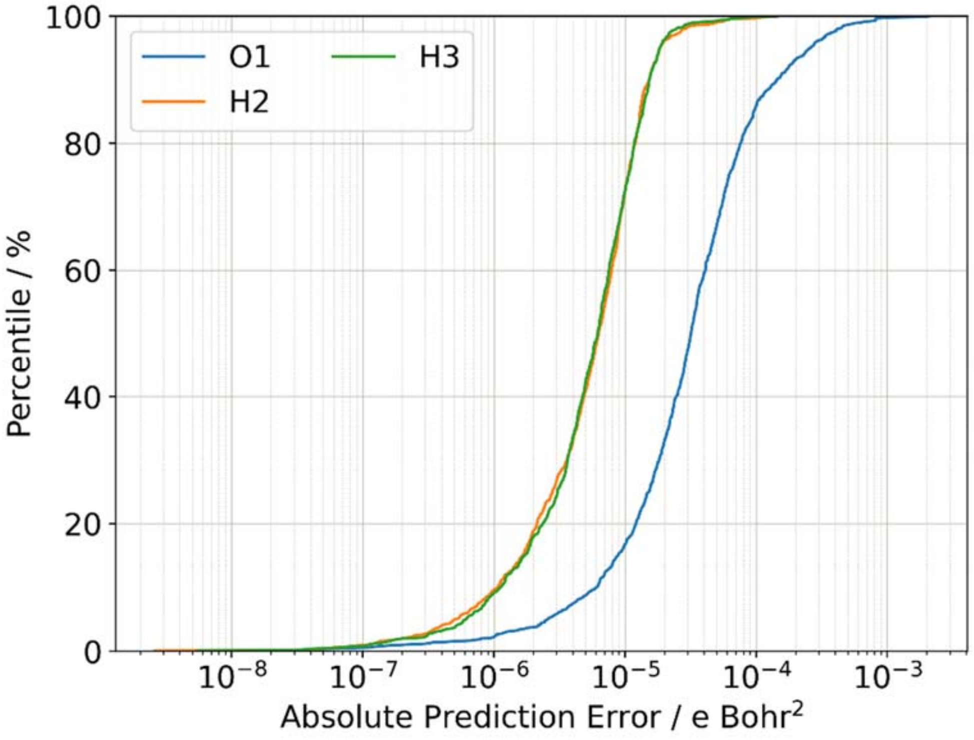

Figure S5 S-curves showing the absolute prediction error in the Q20 component of the atomic quadrupole moment of the water monomer model.

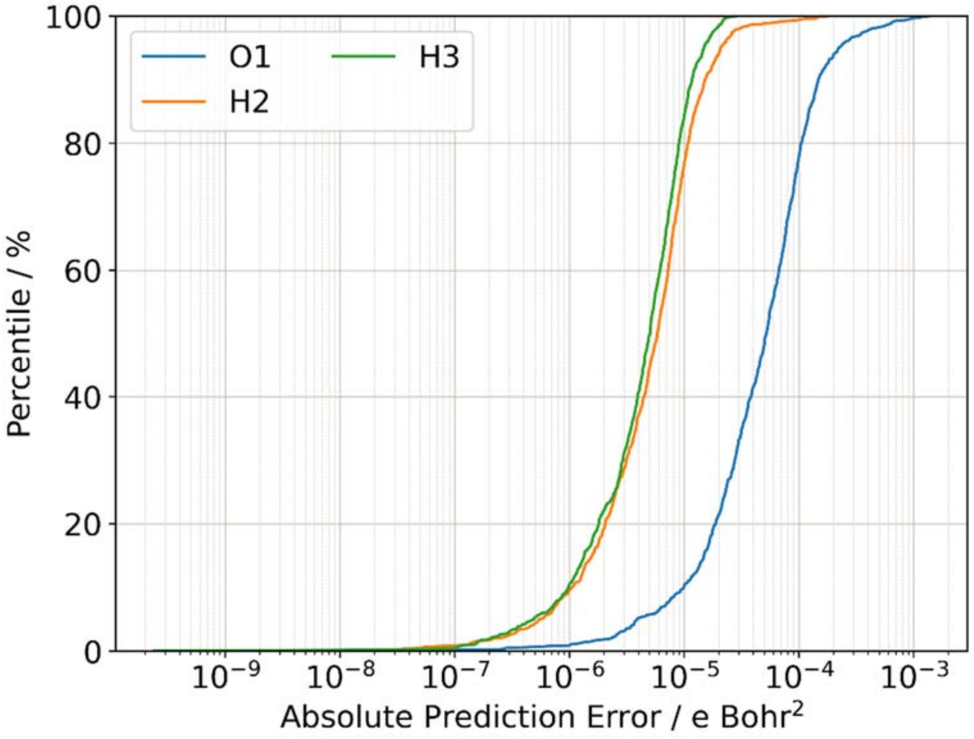

Figure S6 S-curves showing the absolute prediction error in the Q21c component of the atomic quadrupole moment of the water monomer model.

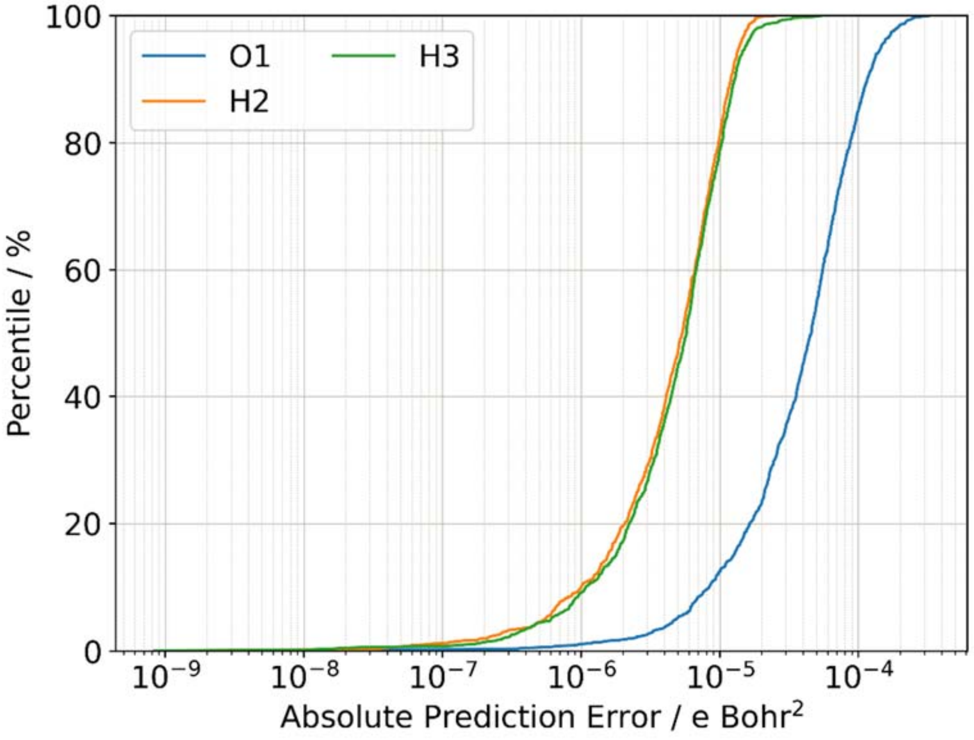

Figure S7 S-curves showing the absolute prediction error in the Q21s component of the atomic quadrupole moment of the water monomer model.

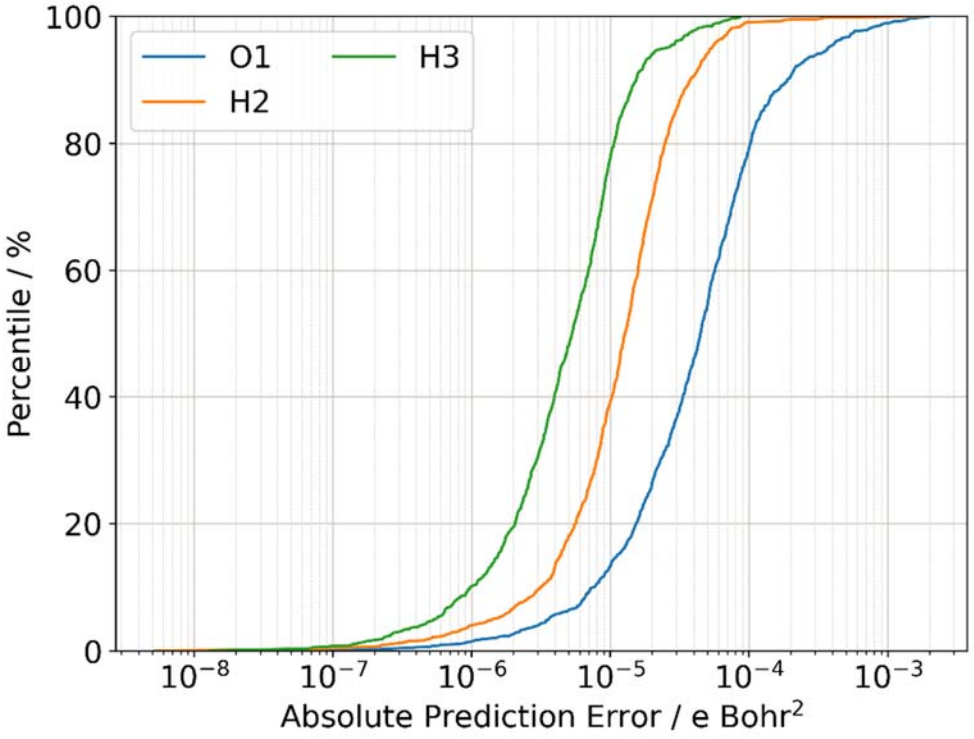

Figure S8 S-curves showing the absolute prediction error in the Q22c component of the atomic quadrupole moment of the water monomer model.

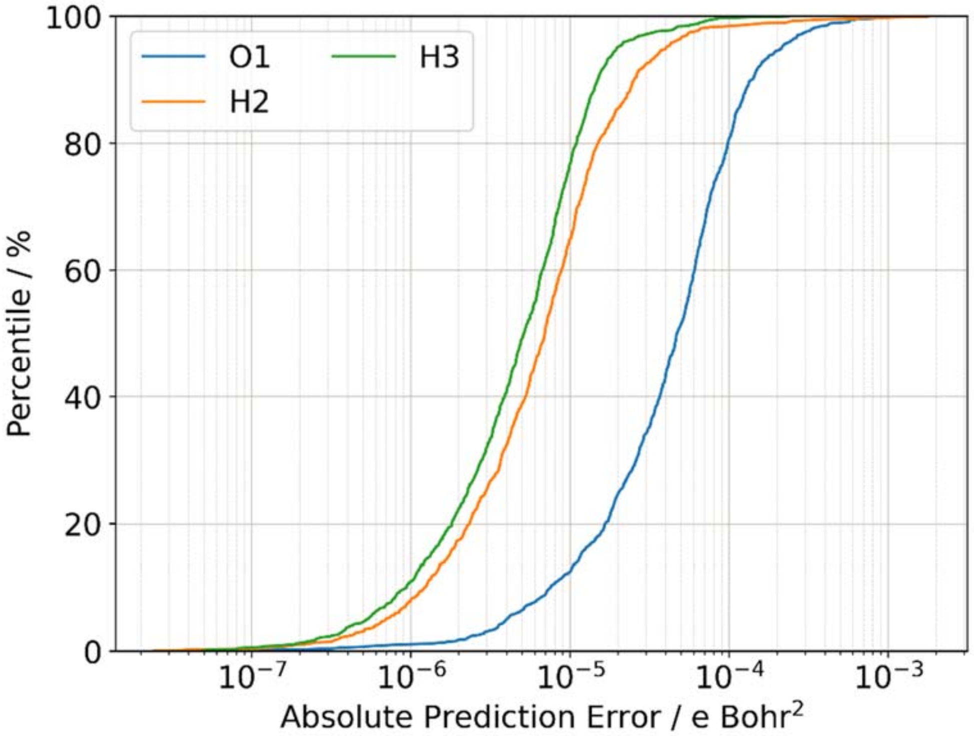

Figure S9 S-curves showing the absolute prediction error in the Q22s component of the atomic quadrupole moment of the water monomer model.

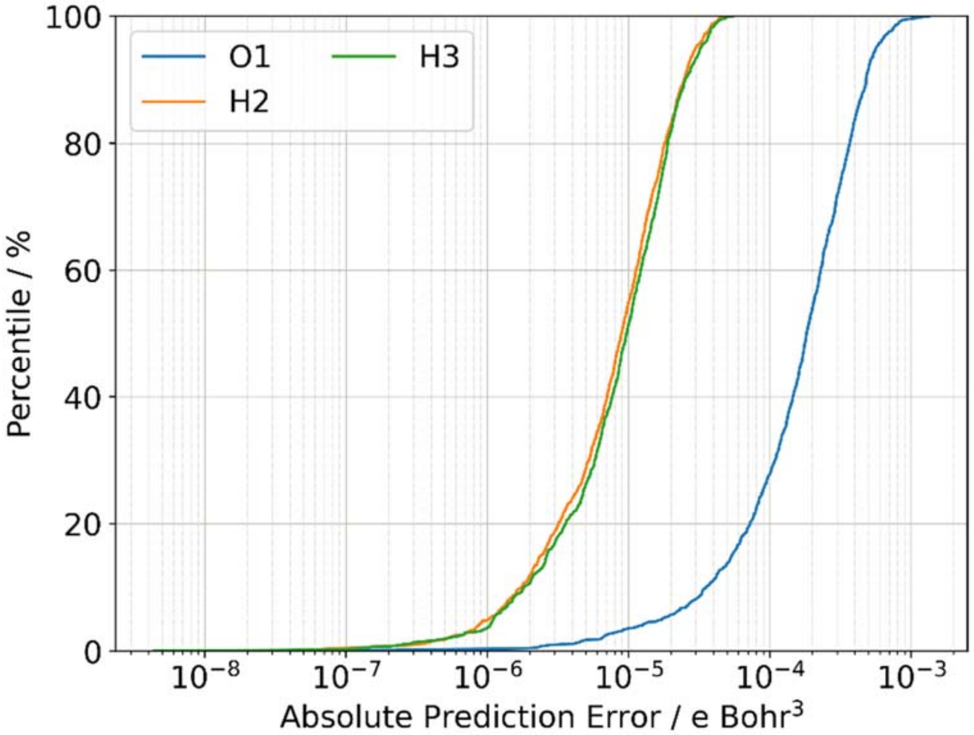

Figure S10 S-curves showing the absolute prediction error in the Q30 component of the atomic octupole moment of the water monomer model.

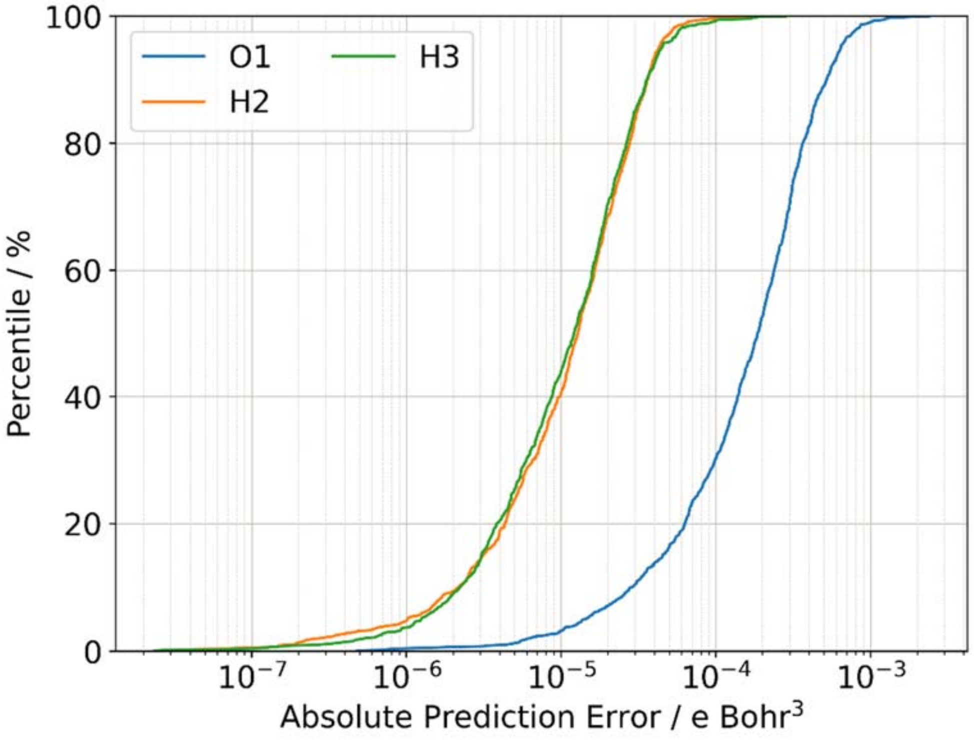

Figure S11 S-curves showing the absolute prediction error in the Q31c component of the atomic octupole moment of the water monomer model.

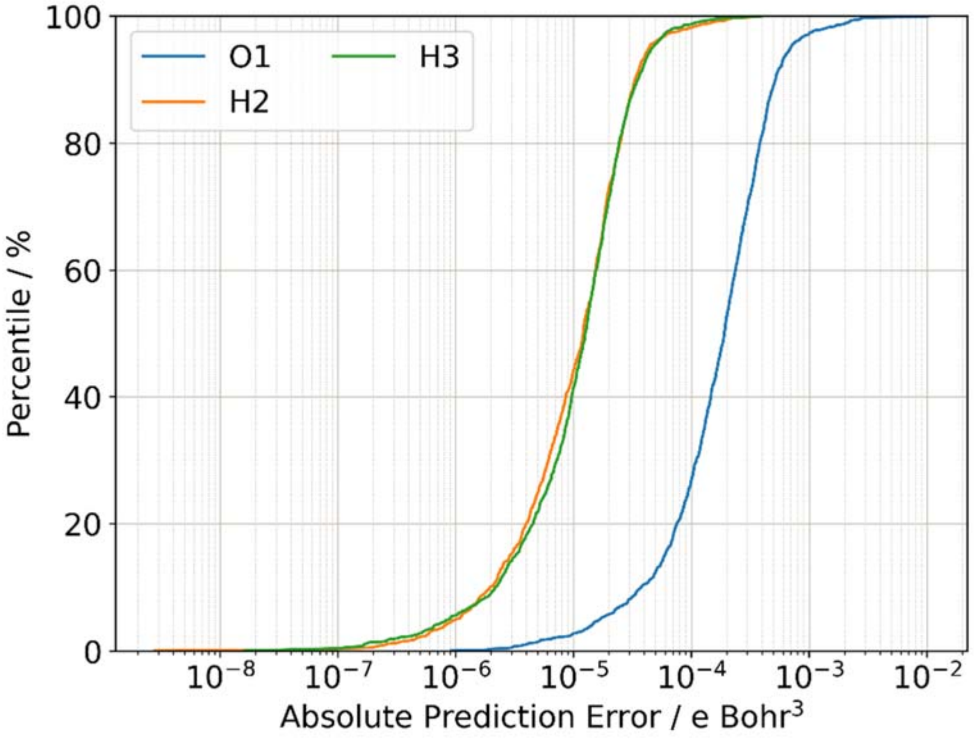

Figure S12 S-curves showing the absolute prediction error in the Q31s component of the atomic octupole moment of the water monomer model.

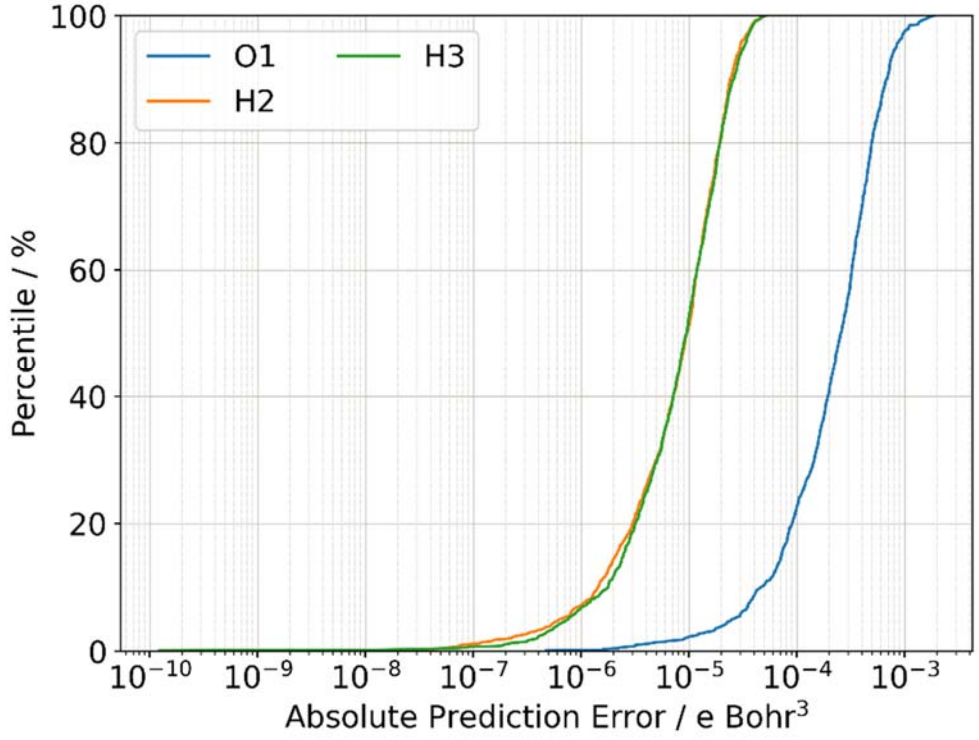

Figure S13 S-curves showing the absolute prediction error in the Q32c component of the atomic octupole moment of the water monomer model.

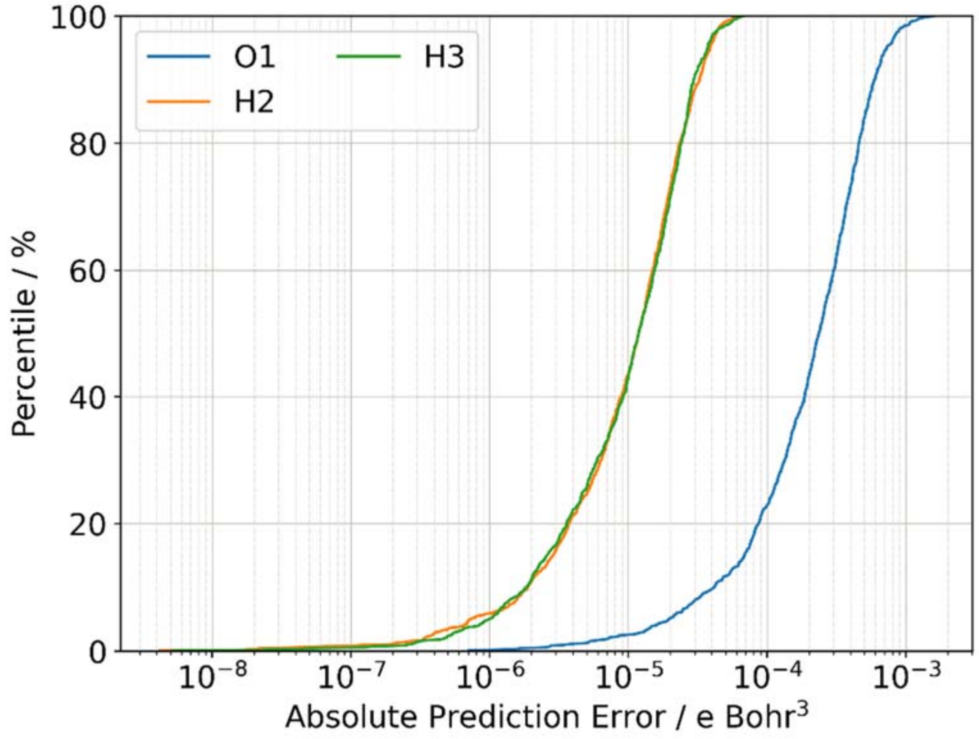

Figure S14 S-curves showing the absolute prediction error in the Q32s component of the atomic octupole moment of the water monomer model.

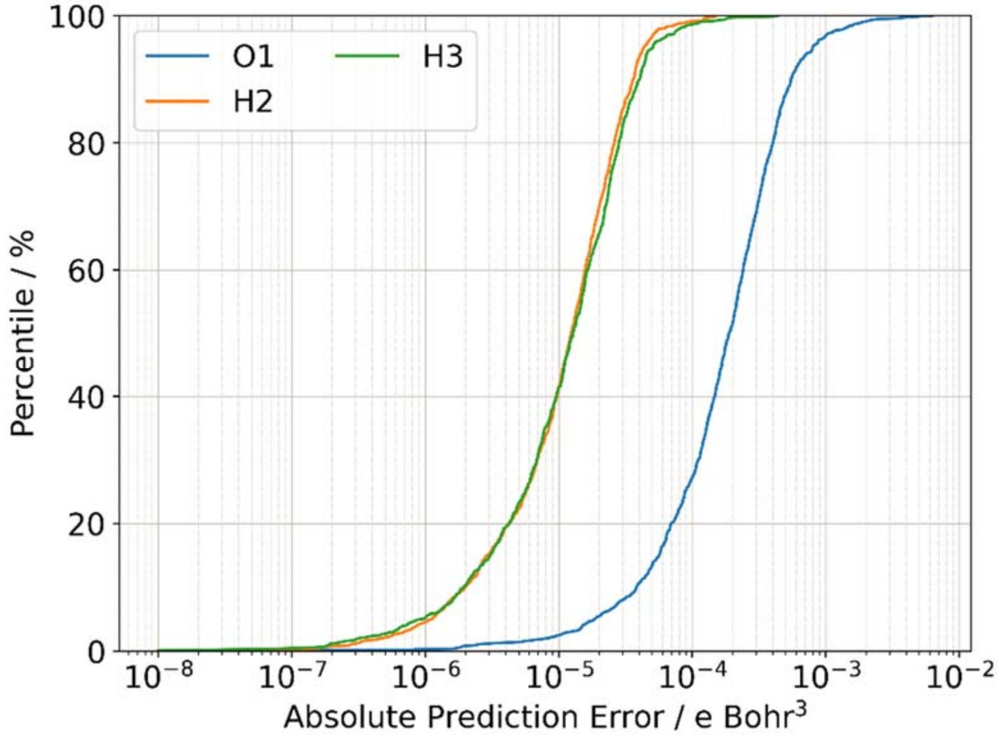

Figure S15 S-curves showing the absolute prediction error in the Q33c component of the atomic octupole moment of the water monomer model.

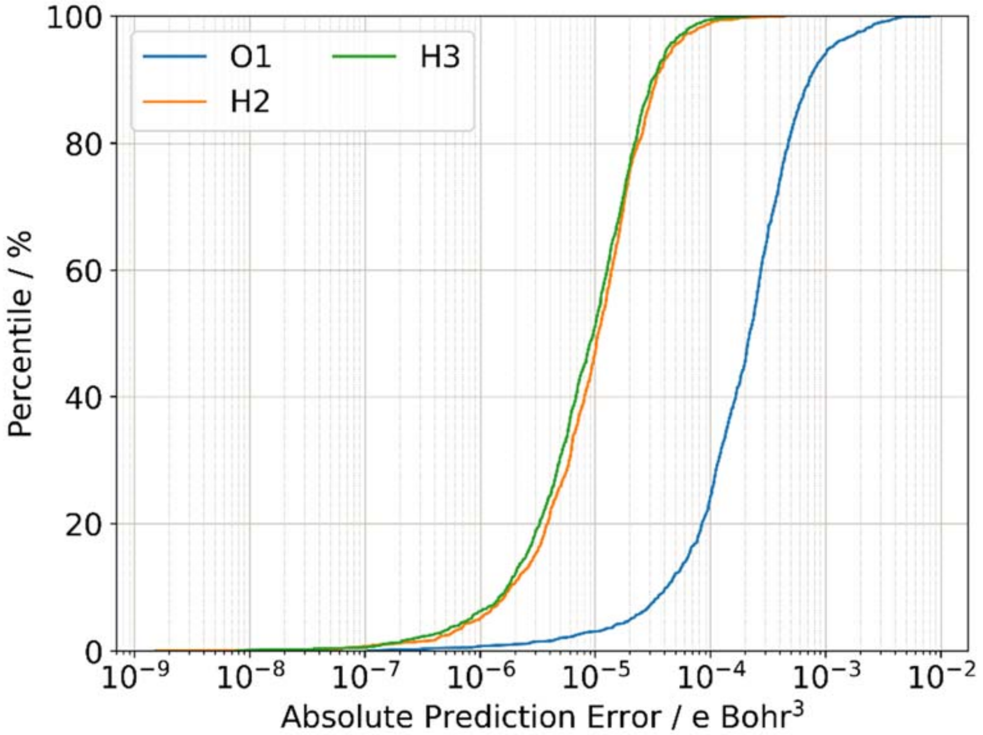

Figure S16 S-curves showing the absolute prediction error in the Q33s component of the atomic octupole moment of the water monomer model.

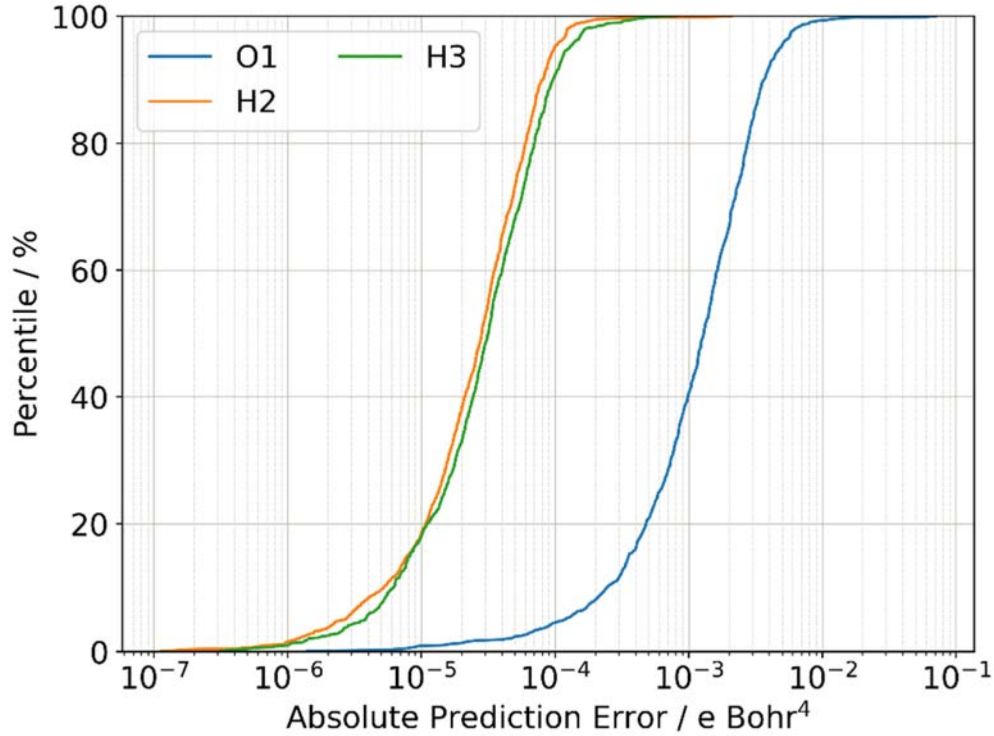

Figure S17 S-curves showing the absolute prediction error in the Q40 component of the atomic hexadecapole moment of the water monomer model.

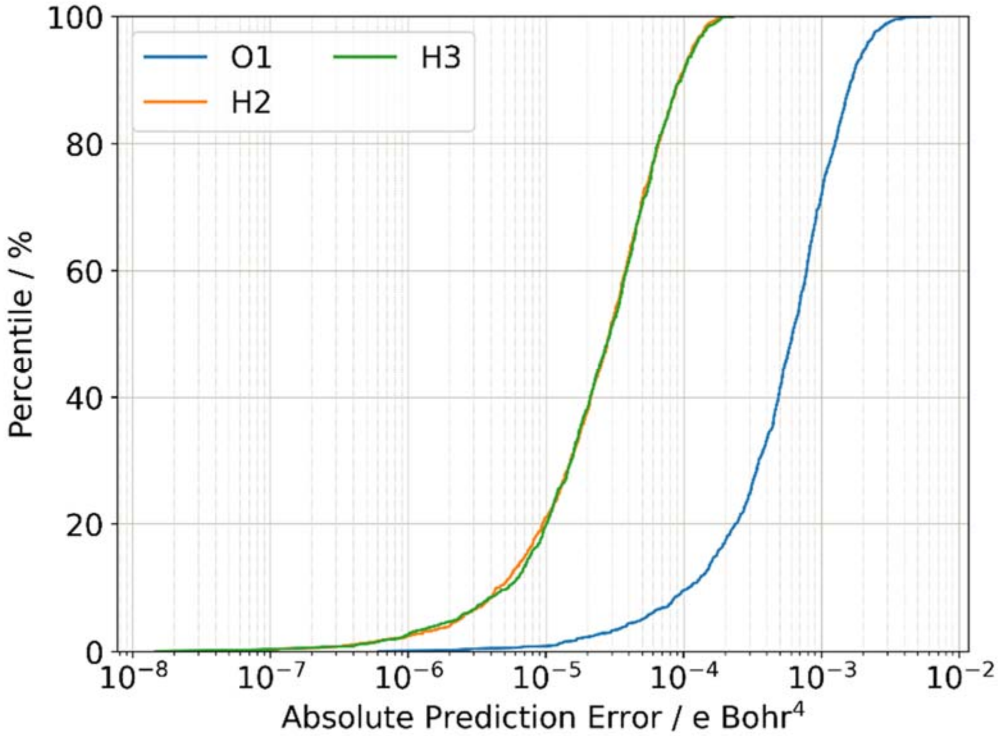

Figure S18 S-curves showing the absolute prediction error in the Q41c component of the atomic hexadecapole moment of the water monomer model.

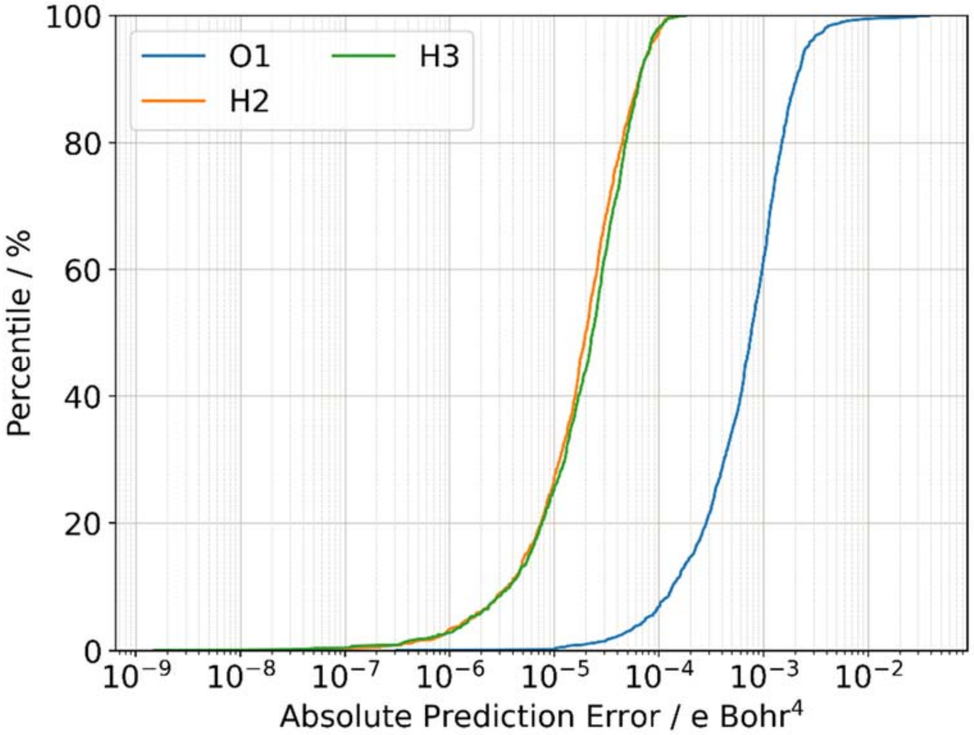

Figure S19 S-curves showing the absolute prediction error in the Q41s component of the atomic hexadecapole moment of the water monomer model.

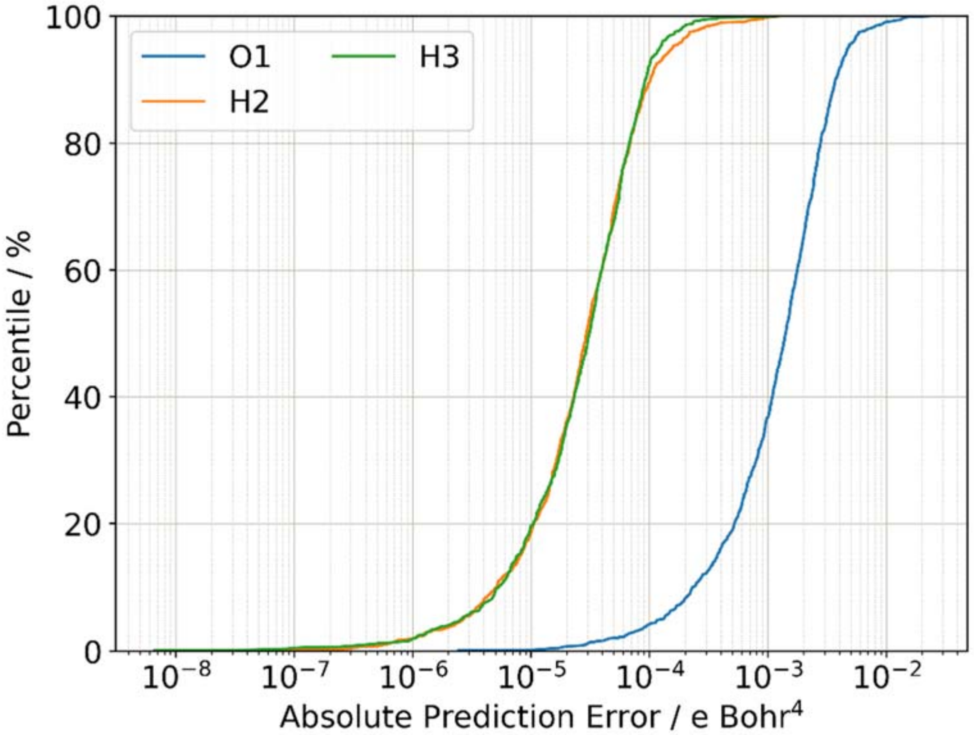

Figure S20 S-curves showing the absolute prediction error in the Q42c component of the atomic hexadecapole moment of the water monomer model.

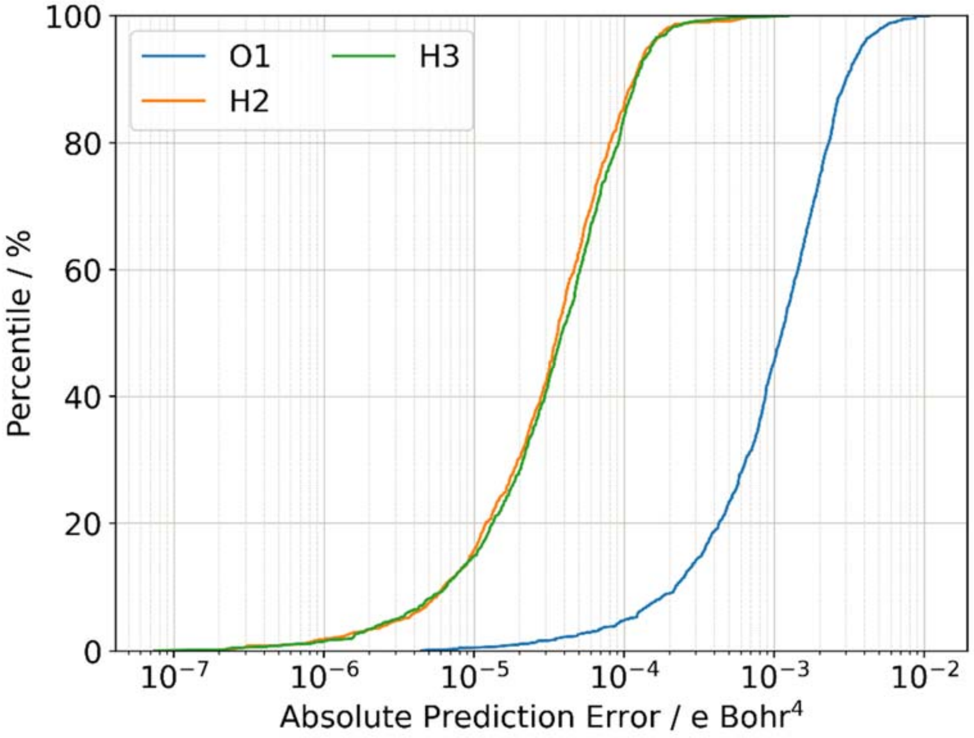

Figure S21 S-curves showing the absolute prediction error in the Q42s component of the atomic hexadecapole moment of the water monomer model.

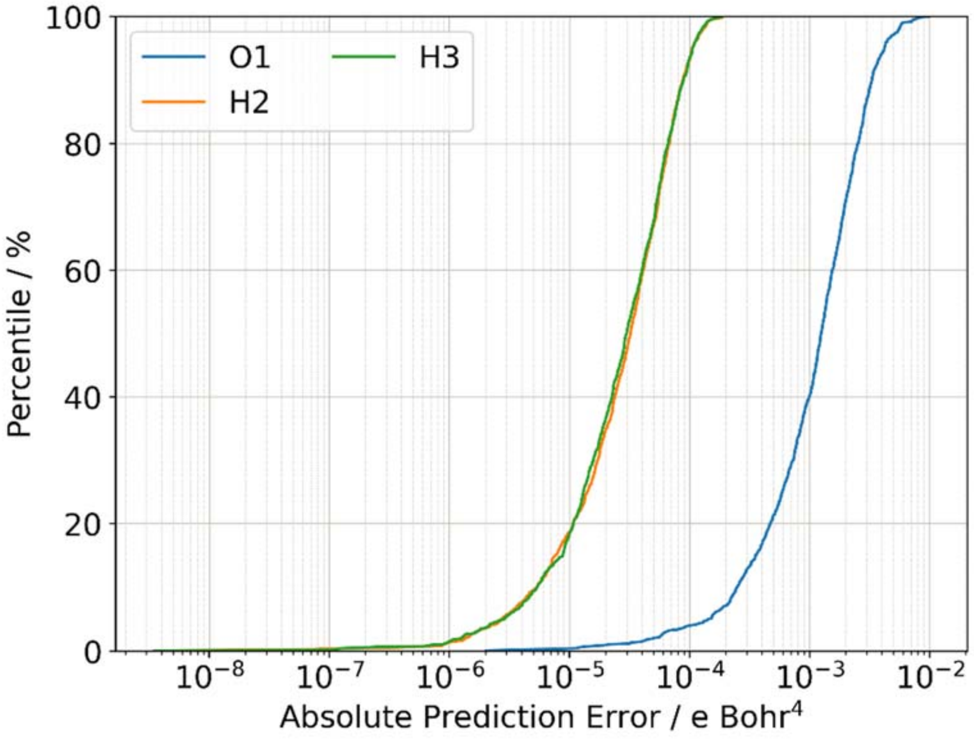

Figure S22 S-curves showing the absolute prediction error in the Q43c component of the atomic hexadecapole moment of the water monomer model.

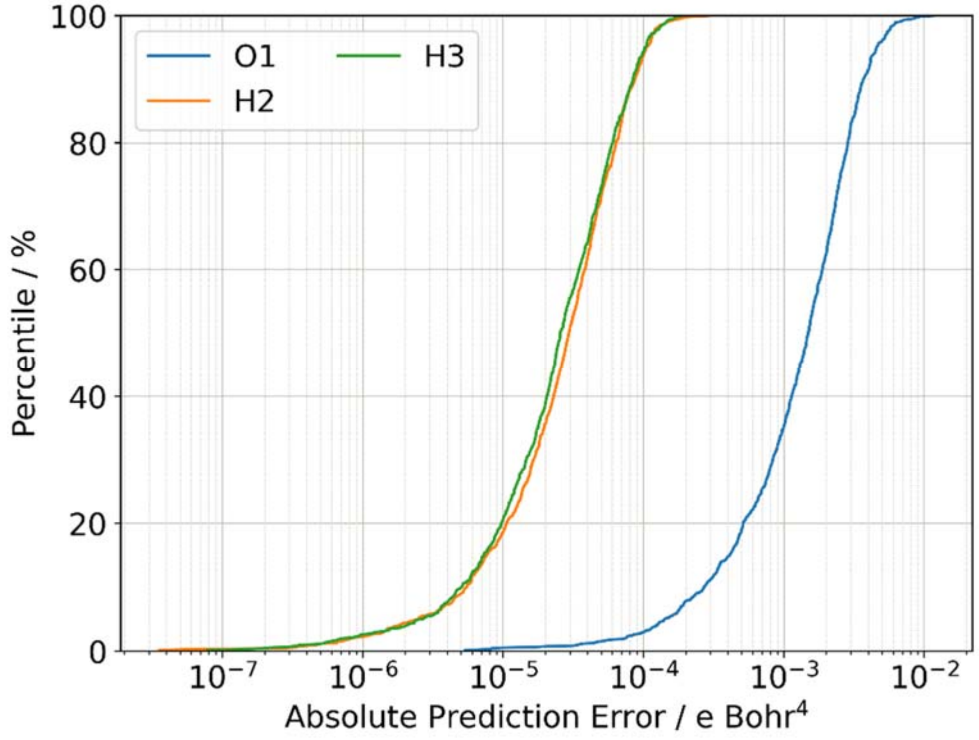

Figure S23 S-curves showing the absolute prediction error in the Q43s component of the atomic hexadecapole moment of the water monomer model.

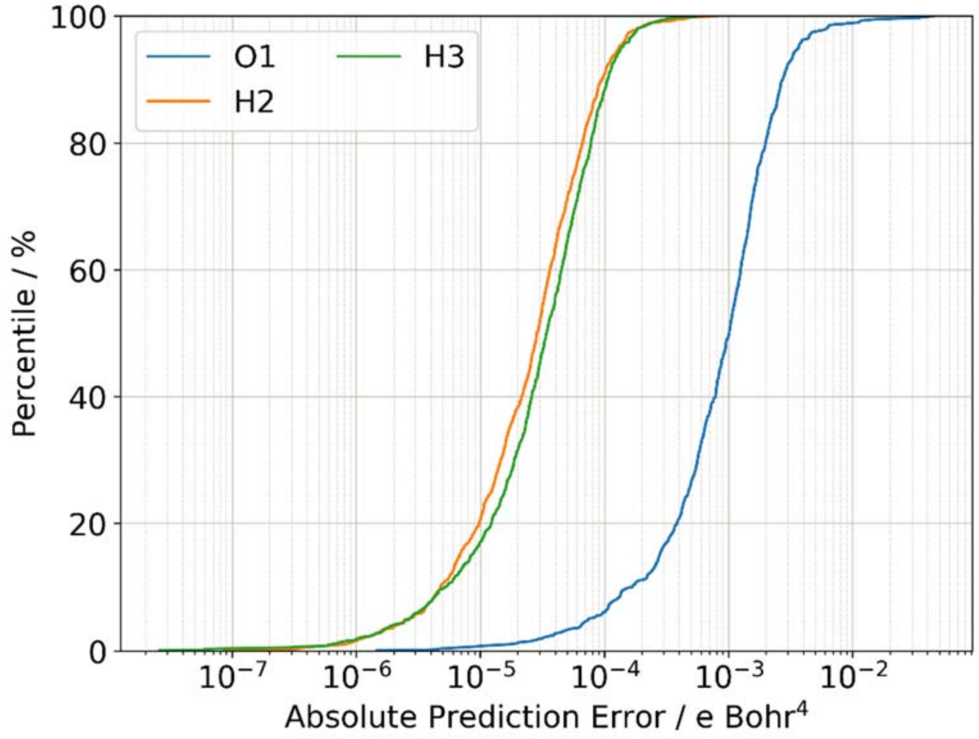

Figure S24 S-curves showing the absolute prediction error in the Q44c component of the atomic hexadecapole moment of the water monomer model.

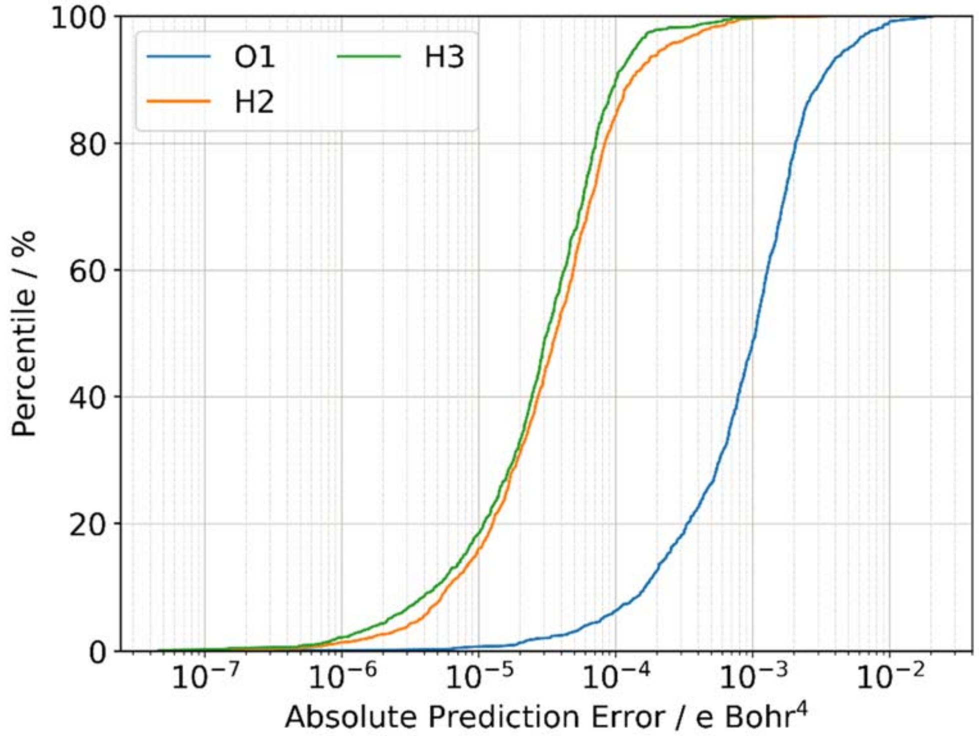

Figure S25 S-curves showing the absolute prediction error in the Q44s component of the atomic hexadecapole moment of the water monomer model.

It is more convenient to represent the errors in multipole moments as electrostatic energies. For this purpose, 500 dimers were generated by combining random points from the 500 K monomer trajectory, placing two molecules between 2-6 Å apart and rotating them randomly along the x, y, and z directions. Wavefunctions were obtained for each monomer using GAUSSIAN09 (Frisch *et al.*, 2010) then parsed into AIMAll to obtain the exact multipole moments for each monomer. The monomeric multipole moments were then used in single-point FFLUX calculations of the dimers to find the "true" electrostatic energies at L' values between 0 and 4 inclusive. The GPR model was then used to predict the electrostatic energies for the dimers and compared to the "true" electrostatic energies. Figure 4 shows errors compared to the L'=4 electrostatic energies as (a) S-curves, and as (b-f) heatmaps showing the RMSE for each interaction across the validation set at each L'.

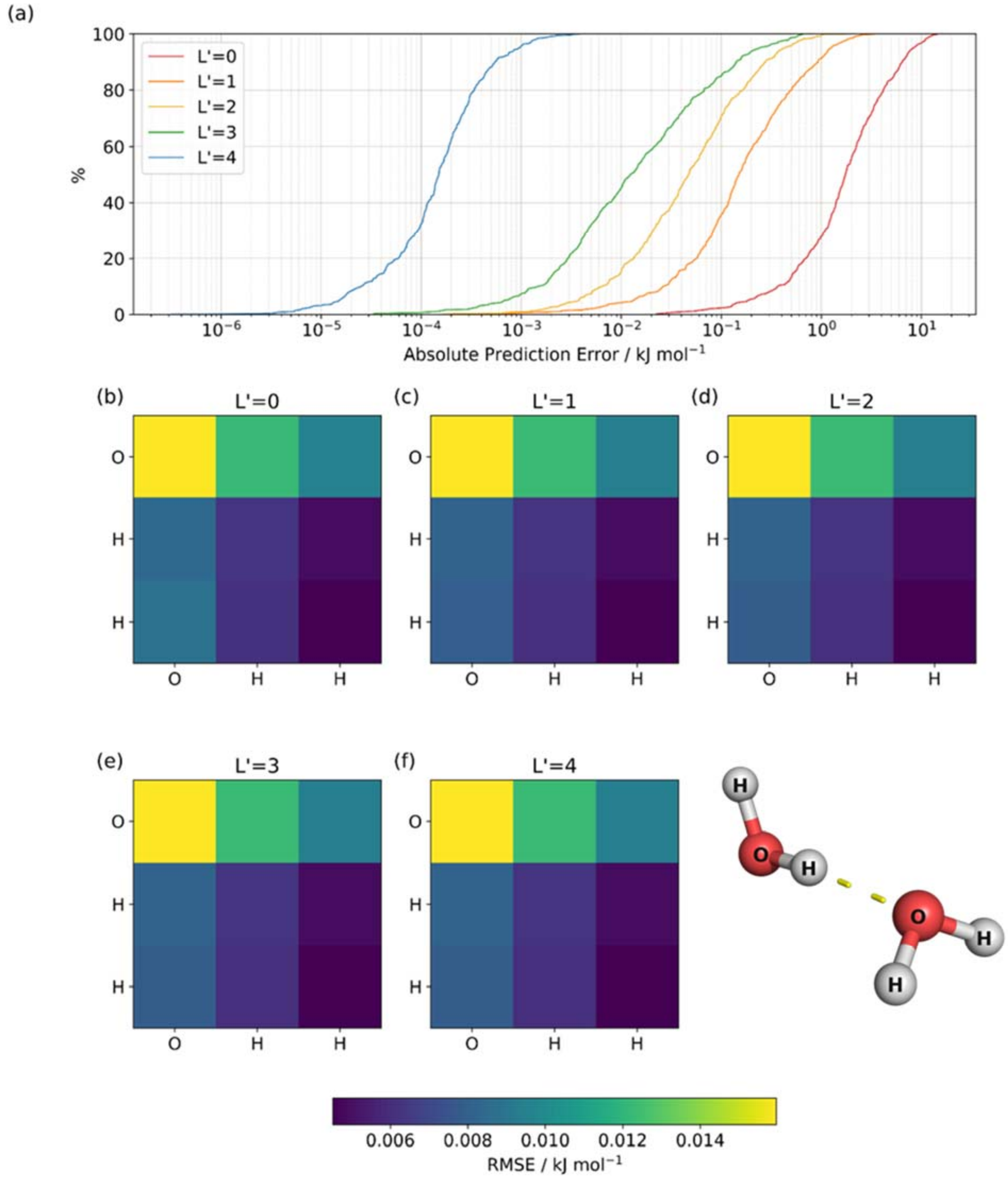

Figure S26 (a) S-curves showing the absolute total electrostatic energy prediction errors for L' ranging between 0 and 4, compared to those calculated with AIMALL at L' = 4. Heatmaps (b-f) show the root-mean-square errors (RMSEs) of the predicted electrostatic energy of each intermolecular atom-atom interaction compared to the electrostatic energy from the AIMAll multipole moments at the same L'.

The S-curves representing prediction errors of the total system electrostatic energy at each L’ are compared to the electrostatic energy calculated using the AIMAll moments calculated at L’=4. Significant improvement in prediction can be seen with each increasing L’ value, with the electrostatic energies predicted at L’=4 showing maximum errors around 3.5 × 10^{-3} kJ mol^{-1}, whilst lower rank multipole moments produce electrostatic energies that differ from the “truth” by nearly 15 kJ mol^{-1}. At L’=2 (the value that was used in crystal calculations), the maximum error in the total electrostatic energy is approximately 1 kJ mol^{-1}, still comfortably below the 4.18 kJ mol^{-1} threshold. The heatmaps show that the O-O interaction contributes the most to these errors at all L’, but the RMSE of this interaction is still very small, around 0.016 kJ mol^{-1}. Moreover, most RMSEs of interactions including hydrogens are below 0.010 kJ mol^{-1}. Overall, the electrostatic energies are predicted with high accuracy by the model. It should be noted though, that while the flexible moments allow for all intramolecular polarisation to be calculated using FFLUX, intermolecular polarisation is not accounted for. The lack of intermolecular polarisation possibly contributes to errors in the ice calculations presented in the main text. While not done in this work, inclusion of polarisation though oligomeric models in FFLUX is possible.

In Section 4.1 of the main text, the GPR model was shown to reproduce the B3LYP/aug-cc-pVTZ optimised geometry with a root-mean-square error of 2.84 × 10^{-4} Å and its energy with an error of only 0.06 kJ mol^{-1}. Additionally, a measure of how well the potential energy surface (PES) is reproduced by the GPR model can be obtained by calculation of vibrational frequencies. These calculations were performed using the finite-difference method implemented in Phonopy (Togo & Tanaka, 2015) (as outlined in Section 3.4) using FFLUX, and compared to vibrational frequencies calculated using B3LYP/aug-cc-pVTZ by GAUSSIAN16 (M. J. Frisch & G. W. Trucks, 2016). The frequencies associated with the three vibrational normal modes of water as calculated by both methods are compared in Table S4.

Table S4 Vibrational frequencies of the water monomer predicted by FFLUX compared to B3LYP/aug-cc-pVTZ, with their differences in frequency (cm^{-1}) and the equivalent energy difference (kJ mol^{-1}).

Assignment	FFLUX (cm^{-1})	B3LYP (cm^{-1})	Difference (cm^{-1})	Energy difference (kJ mol^{-1})
HOH bend	1629.41	1627.77	1.67	0.020
O-H symmetric stretch	3797.83	3797.04	0.79	0.009
O-H asymmetric stretch	3906.26	3899.28	6.98	0.083

FFLUX reproduces the vibrational frequencies with a maximum error of 6.98 cm^{-1} in the asymmetric OH stretch, corresponding to a maximum energy error that is orders of magnitude lower than the 4.18 kJ mol^{-1} threshold of chemical accuracy. This indicates that the potential energy surface of the training level of theory is reproduced well.

The stability of the GPR model during molecular dynamics simulations is another important metric to consider when assessing the performance of a model, sometimes referred to as a robustness test. To investigate the stability of the water monomer model, MD simulations were performed at temperatures between 50 and 500 K, with five runs at each temperature (50 K, 100 K, then in steps of 100 K up to 500 K). Three different seed numbers were added to each CONTROL file randomly, which ensures that no runs will have identical initial velocities and therefore identical trajectories. Simulations were conducted in the *NVT* ensemble using the Nosé-Hoover thermostat (with a relaxation time of 0.01 ps) for 5 million steps with a 1 fs time step corresponding to 5 ns. Bond lengths were measured at every step in the trajectories and the difference from the B3LYP/aug-cc-pVTZ reference structure was calculated. The model was shown to be stable for 5 ns at every temperature by verifying that none of the differences are above a certain threshold based on a literature value of 0.5 Å (Fu *et al.*, 2023). While this threshold has been used in other work, it is not necessarily appropriate for water, representing a change of 52% in bond length. Therefore, several smaller thresholds were tested, with the smallest threshold that maintained the 5 ns stability being 0.09 Å, meaning no bonds differed by a value larger than that compared to the reference.

S4. Harmonic approximation

S4.1. Phonon dispersion curves

The dynamical stability of structures can be investigated through the phonon dispersion. The phonon dispersion curves of FFLUX-optimised structures I*h* and XV are shown in Figure S27.

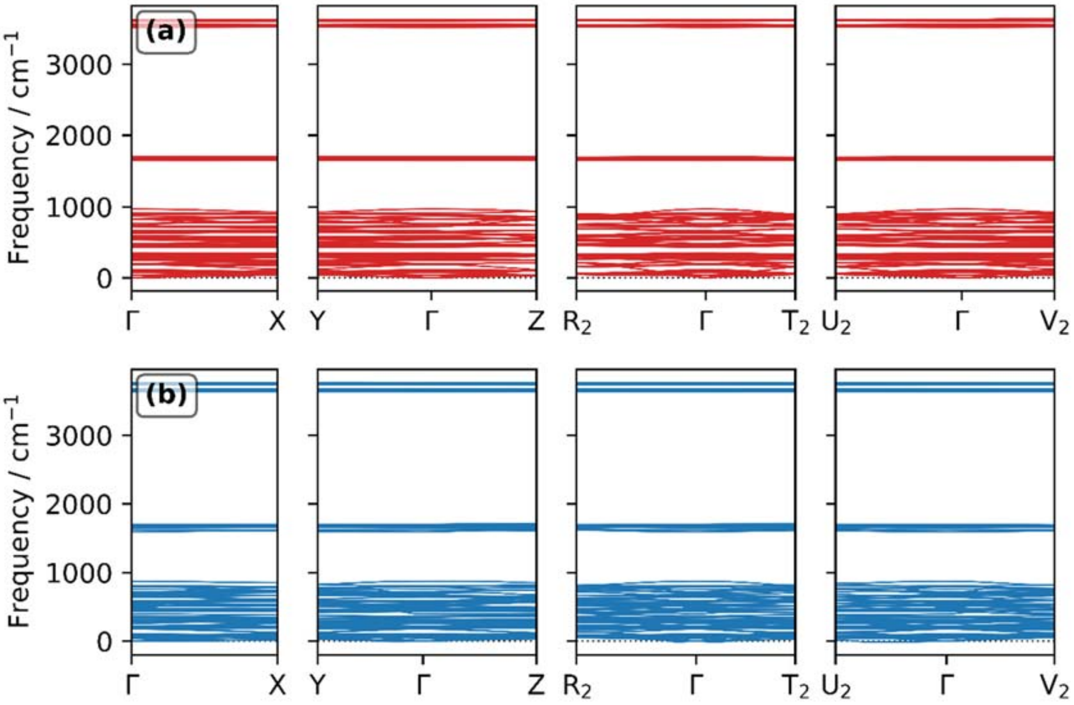

Figure S27 Phonon dispersion curve of (a) I*h* and (b) XV calculated by FFLUX.

In the dispersion of both I*h* and XV, two types of imaginary modes can be observed: firstly, the negative frequencies present at point Γ, which correspond to acoustic modes. These modes are due to translational and rotational motion and do not indicate dynamical instability. In this case, they were on the order of 10^{-3} THz. Secondly, imaginary frequencies appear between points Γ and *U2*, but not directly at any high-symmetry points. These are artefacts of Fourier interpolation (Pallikara *et al.*, 2022), which often disappear at increased supercell expansions and can thus be ignored in the evaluation of dynamical stability. Therefore, it can be safely assumed that the FFLUX optimised structures of ice I*h* and XV are dynamically stable. The stability of the phases was also assessed in the PBE+D3 calculations with the calculated phonon dispersions of all phases shown in Figure S28.

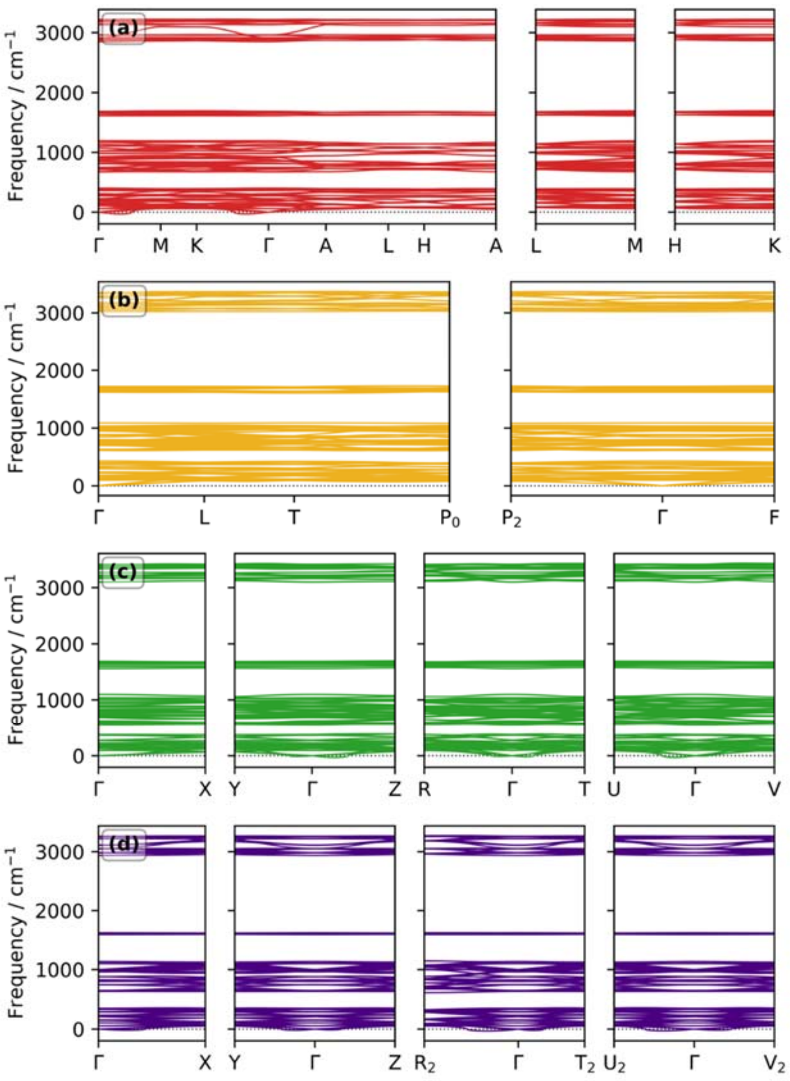

Figure S28 Phonon dispersions of ice (a) I*h*, (b) II, (c) XV, and (d) II' calculated using PBE+D3.

S4.2. Ice II'

The phonon dispersion of phase II using FFLUX contained imaginary phonon modes, indicating dynamical instability. This instability was investigated by applying the 'mode-mapping' technique using the ModeMap code (Skelton *et al.*, 2016). The process involves generating distorted structures along the imaginary mode eigenvectors. Displacements were generated with a displacement amplitude (Q) range between -20 to 20 amu$^{1/2}$ Å in steps of 0.5 amu$^{1/2}$ Å along all wavevectors with imaginary modes (*X, Z,* R_2) using a 6 × 6 × 6 supercell expansion. When the original 5 × 5 × 5 supercell was used, the mapped energies gave a harmonic curve, which would indicate that it is an equilibrium structure, contradicting the imaginary modes. However, using a 6 × 6 × 6 expansion the expected curve was observed. Single-point force calculations were performed on each structure to plot the PES. The PESs mapped along wavevectors X and Z are shown in Figure S29 and S30.

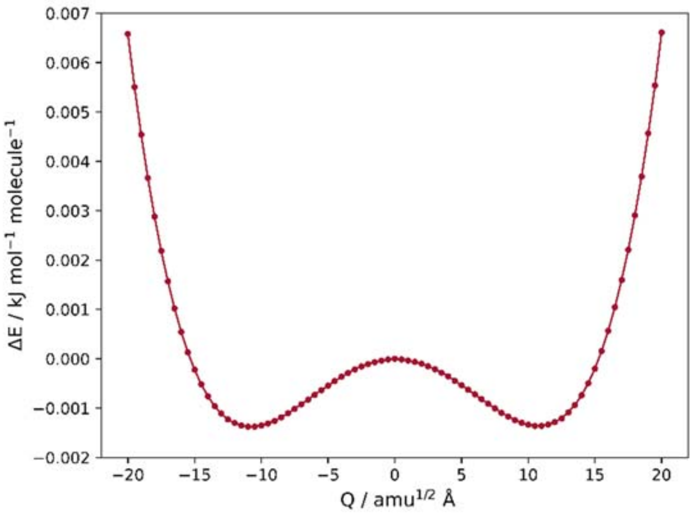

Figure S29 Anharmonic double-well PES along wavevector X.

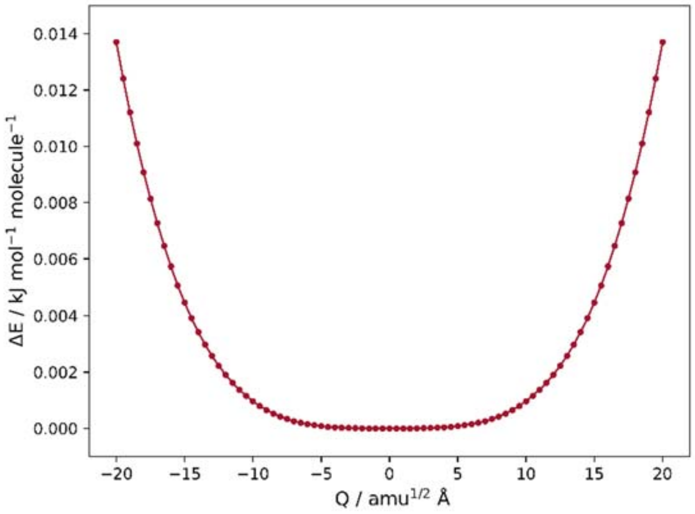

Figure S30 Harmonic PES along wavevector Z.

The mapped PES (Figure S30) was harmonic despite the doubly degenerate negative frequencies seen at wavevector Z. This discrepancy is likely due to numerical errors in the force calculations caused by incorrect non-bonded parameters.

The point with the most negative frequency, point R_2, was considered for selecting the lowest energy structure on the PES. The minimum energy structure generated from the displacements was then optimised in a 7000 step $N\sigma T$ run using the same settings as described in Section 3.2. The converged structure was determined as detailed in the same section. This structure, labelled II', differs from the known phase II as demonstrated below.

To see if II’ is stable outside of FFLUX parametrisation, the structure was optimised using PBE+D3 and the phonon frequencies were calculated using the same setting as for ice II in Section 3.3. The phonon dispersion curve obtained using PBE+D3 is shown in Figure S28 (d).

Figures S31-S33 show 2 × 2 × 2 supercell expansions of phase II as obtained from experiment (Kamb *et al.*, 2003), and II’ as optimised by FFLUX viewed along axes *a, b* and *c* using VESTA (Momma & Izumi, 2008). Lattice parameters of phase II’ calculated by FFLUX and PBE+D3 are presented in Table S5.

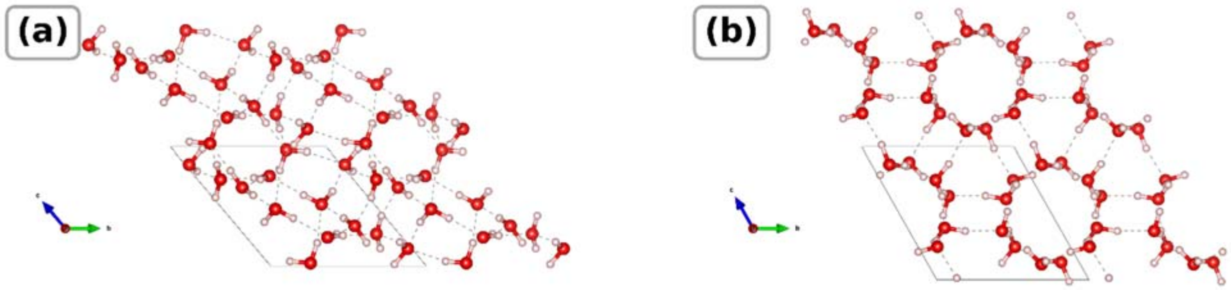

Figure S31 (a) Phase II and (b) II’ viewed along the a-axis.

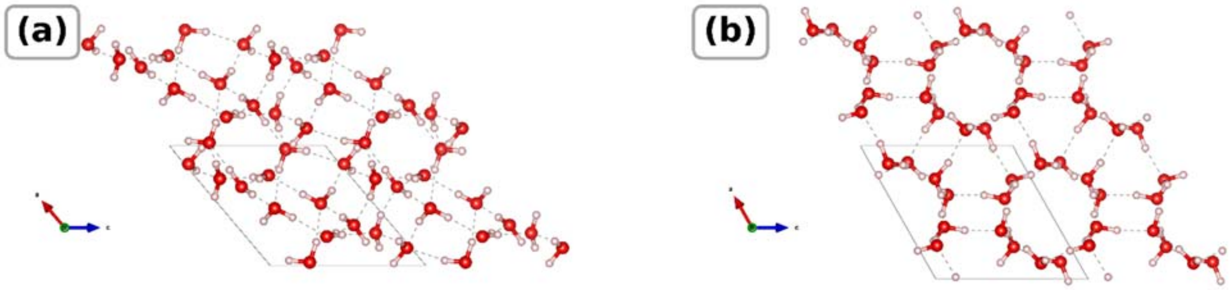

Figure S32 (a) Phase II and (b) II’ viewed along the b-axis.

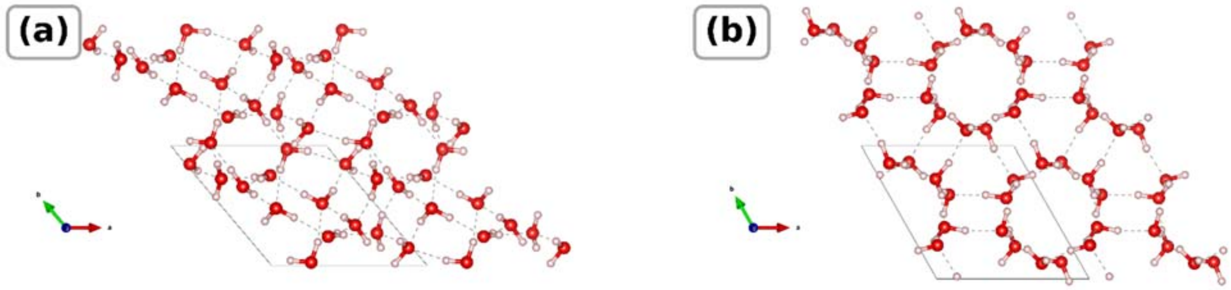

Figure S33 (a) Phase II and (b) II’ viewed along the c-axis.

Table S5 Lattice parameters of II' calculated by FFLUX and PBE+D3 compared to the experimental parameters of ice II (Kamb *et al.*, 2003).

Properties	Experimental Ice II	PBE+D3 Ice II'	FFLUX Ice II'	%Δ PBE+D3 – FFLUX
a (Å)	7.78	7.55	7.60	0.51
b (Å)	7.78	7.55	7.59	0.16
c (Å)	7.78	7.55	7.58	0.21
α (°)	113.10	109.43	109.43	-0.16
β (°)	113.10	109.46	109.45	-0.07
γ (°)	113.10	109.45	109.53	0.01

To further assess the stability of ice II’, phonon calculations within the quasi-harmonic approximation were performed as described in the main text, giving access to Gibbs free energies. The stability of phases II, XV and II’ relative to ice I*h* , and calculated using periodic plane wave PBE+D3, was assessed at pressures between 0 and 10 GPa at a constant temperature of 100 K as shown in Figure S34 .

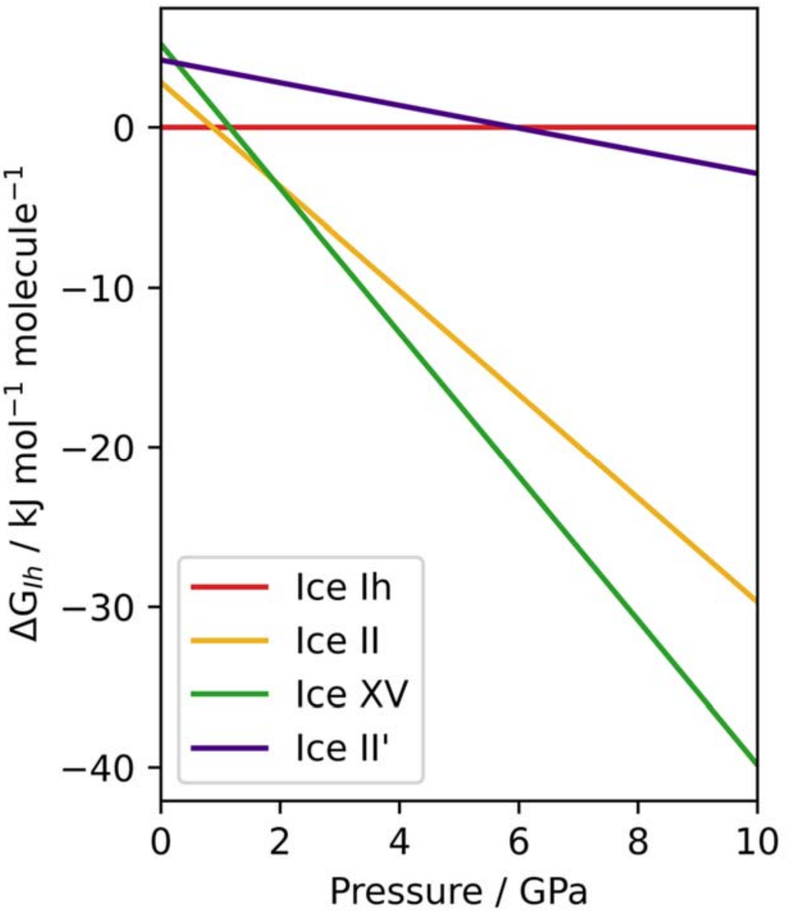

Figure S34 Gibbs free energy of ices II (yellow), XV (green) and II’ (purple) relative to ice Ih (red) across a pressure range of 0 to 10 GPa at 100 K. Calculations were performed using periodic plane wave PBE+D3.

At no point across this pressure range does Ice II’ become the most stable, suggesting that it is a metastable phase.

References

Abascal, J. L. F., Sanz, E., García Fernández, R. & Vega, C. (2005). *J. Chem. Phys.* **122**.

Brown, M. L., Isamura, B. K., Skelton, J. M. & Popelier, P. L. A. (2024). *J. Chem. Theory Comput.* **20**, 5994-6008.

Brown, M. L., Skelton, J. M. & Popelier, P. L. A. (2023). *J. Chem. Theory Comput.* **19**, 7946-7959.

Frisch, M. J., Trucks, G. W., Schlegel, H. B., Scuseria, G. E., Robb, M. A., Cheeseman, J. R., Scalmani, G., Barone, V., Mennucci, B. & Petersson, G. A. (2010). *Gaussian Inc., Wallingford, CT, USA*.

Fu, X., Wu, Z., Wang, W., Xie, T., Keten, S., Gomez-Bombarelli, R. & Jaakkola, T. (2023). *Transactions on Machine Learning Research*.

Hédin, F., El Hage, K. & Meuwly, M. (2016). *J. Chem. Inf. Model.* **56**, 1479-1489.

Kamb, B., Hamilton, W. C., LaPlaca, S. J. & Prakash, A. (2003). *J. Chem. Phys.* **55**, 1934-1945.

M. J. Frisch & G. W. Trucks, H. B. S., G. E. Scuseria, M. A. Robb, J. R. Cheeseman, G. Scalmani, V. Barone, G. A. Petersson, H. Nakatsuji, X. Li, M. Caricato, A. V. Marenich, J. Bloino, B. G. Janesko, R. Gomperts, B. Mennucci, H. P. Hratchian, J. V. Ortiz, A. F. Izmaylov, J. L. Sonnenberg, Williams, F. Ding, F. Lipparini, F. Egidi, J. Goings, B. Peng, A. Petrone, T. Henderson, D. Ranasinghe, V. G. Zakrzewski, J. Gao, N. Rega, G. Zheng, W. Liang, M. Hada, M. Ehara, K. Toyota, R. Fukuda, J. Hasegawa, M. Ishida, T. Nakajima, Y. Honda, O. Kitao, H. Nakai, T. Vreven, K. Throssell, J. A. Montgomery Jr., J. E. Peralta, F. Ogliaro, M. J. Bearpark, J. J. Heyd, E. N. Brothers, K. N. Kudin, V. N. Staroverov, T. A. Keith, R. Kobayashi, J. Normand, K. Raghavachari, A. P. Rendell, J. C. Burant, S. S. Iyengar, J. Tomasi, M. Cossi, J. M. Millam, M. Klene, C. Adamo, R. Cammi, J. W. Ochterski, R. L. Martin, K. Morokuma, O. Farkas, J. B. Foresman and D. J. Fox (2016). *GAUSSIAN16*.

McDonagh, J. L., Silva, A. F., Vincent, M. A. & Popelier, P. L. A. (2018). *J. Chem. Theory Comput.* **14**, 216−224.

Momma, K. & Izumi, F. (2008). *J. Appl. Crystallogr.* **41**, 653-658.

Pallikara, I., Kayastha, P., Skelton, J. M. & Whalley, L. D. (2022). *Electron. Struct.* **4**, 033002.

Skelton, J. M., Burton, L. A., Parker, S. C., Walsh, A., Kim, C.-E., Soon, A., Buckeridge, J., Sokol, A. A., Catlow, C. R. A., Togo, A. & Tanaka, I. (2016). *Phys. Rev. Lett.* **117**, 075502.

Togo, A. & Tanaka, I. (2015). *Scr. Mater.* **108**, 1-5.
